# Stakeholders’ perspectives on barriers to and facilitators of school-based HPV vaccination in the context of COVID-19 pandemic-related disruption: a qualitative mixed methods study

**DOI:** 10.1080/17482631.2023.2295879

**Published:** 2023-12-20

**Authors:** Amal Khan, Sylvia Abonyi, Cory Neudorf, Sandro Galea, Shahid Ahmed

**Affiliations:** a Department of Community Health and Epidemiology, University of Saskatchewan, Saskatoon, Canada; b Public Health, Boston University School of Public Health, Boston, MA, USA; c Department of Medical Oncology, Saskatoon Cancer Center, Saskatchewan Cancer Agency, Saskatoon, Canada

**Keywords:** HPV vaccine, human papillomavirus vaccination, preventable cancers, stakeholders, providers, system level workers, HPV vaccine uptake factors, COVID-19 pandemic related disruptions, school-based HPV immunization programme, barriers and facilitators

## Abstract

Despite successfully implementing the Human Papilloma Virus Vaccine (HPVV) program, Saskatchewan (SK) struggled to improve HPVV uptake rates. This suboptimal uptake of HPVV with a status quo of HPV-linked cervical cancer incidence rate is mainly because HPVV’s impact on cancer prevention has not been realized adequately by vaccine providers and receivers. Further exploration of determinants of HPVV uptake is required to uncover high-resolution quality improvement targets for investment and situate contextually appropriate policies to improve its uptake. The study undertook a qualitative inquiry into understanding stakeholders’ perspectives on HPVV experience through school-based programmes. It collected data through semi-structured initial interviews (N = 16) and follow-up interviews (N = 10) from across Saskatchewan’s four Integrated Service Areas. Document analysis was conducted on all publicly available documents that included information on HPVV from January 2015 to July 2023. Thematic analysis of the data identified that inadequate information, awareness and education about HPV infection and HPVV among several groups, especially, parents, youth and school staff, was the main barrier to optimal HPVV uptake. Vaccine-related logistics, including the technical and text-heavy vaccine information sheet, understaffing, and time constraints, were other important factors that impeded HPVV uptake. A person-centred approach could educate parents in multiple dimensions.

## Introduction

HPV is the commonest Sexually Transmitted Infection (STI) around the globe (Cervical cancer, 2023) and is responsible for more than 95% of cervical cancers (Cervical cancer, 2023; de Martel et al., 2017). HPV infection increases the risk of squamous cell cancer of various sites, including cancers of the cervix, vagina, vulva, penis, anus and head and neck region (Cancers associated with human Papillomavirus HPV, 2022). More than 75% of sexually active Canadians will contract at least one HPV infection in their lifetime (Canadian Cancer Society, Statistics Canada, the Public Health Agency of Canada, 2020). Human Papillomavirus Virus (HPV) related cancers can largely be prevented by vaccination (Canadian Cancer Society’s Advisory Committee on Cancer Statistics, 2016). Oncosims model projections indicate that higher uptake rates of Human Papilloma Virus Vaccination (HPVV) from 67% to 90% would result in a 23% reduction in incidence rates of cervical cancer hence causing a 23% decline in the cervical cancer mortality rate on average annually (Smith et al., 2019).

Canada has been a success story in launching and operating a school-based HPVV programme (Canadian Cancer Society’s Advisory Committee on Cancer Statistics, 2016). Despite the wide availability of HPVV through publicly funded programmes, in Canada, there are 3,800 (2022) new cancer cases annually attributed to HPV (reported in 2021), and according to the estimates, this number will reach 6600 by 2024 (HPV immunization for the prevention of cervical cancer, 2021). Saskatchewan (SK) successfully launched HPVV school-based programme for girls (in grades 6 and 7) in 2008 (Canadian Partnership Against Cancer, 2021; Population health branch, Saskatchewan ministry of health, 2019) and expanded it to include the vaccination of boys in grades 6 in the fall of 2017 (Human papilloma virus HPV vaccine offered to boys beginning this fall, 2023; Population health branch, Saskatchewan ministry of health, 2019).

Despite successfully implementing the HPVV programme, SK struggled to improve its HPVV uptake rates. In the HPVV programme’s first year (2008–2009), the HPVV uptake rate was 74.5%, transiently increasing to 76.6% before consistently dropping to as low as 61.4% (2014/15). A report (2021) indicated that SK remains below the national target for HPVV uptake and is amongst the provinces that have “the lowest” uptake rates (Background and key statistics, 2021). It is likely that the underutilization of HPVV in some Canadian jurisdictions is directly contributing to the HPV related cancers burden in Canada.

We, therefore, undertook a project that first determined the current state of science on the factors that influence the uptake of HPV vaccine across English Canada (Khan et al., 2023) and then explored people’s perspectives on the HPVV uptake through school-based programmes across SK at three levels: patients-, providers- and system -level. We also determined the COVID-19 pandemic-related disruption of the school-based HPVV programme across SK regarding the problem, lessons learnt, and current mandate to reach HPV vaccine coverage targets. This paper reports on our exploration of the system and provider level (stakeholders) perspective on the uptake of HPVV across SK.

## Methods

### Study design

The project employed Qualitative Sequential Mixed Methods (Morse, 2010) adopting an Interpretive Description (Thorne, 2016) approach. Engel’s Bio-Psyco-Social lens (Borrell-Carrió et al., 2004) serves as the theoretical underpinning of the project, with pragmatism as the project’s philosophy. This paper reports on one aspect of our study that focuses on our exploration of *stakeholders’ perspectives* on barriers to and facilitators of HPVV uptake through school-based vaccine programmes from across SK. The study also determined the COVID-19 Pandemic impact on school-based HPVV programmes across SK in terms of the scope of the problem posed and the current mandate to reach the HPV vaccine coverage targets. The following research questions guided the study design:

1. What are providers’ (supervisors and frontline workers) perspectives on provider-level barriers to and facilitators of HPV vaccination across Saskatchewan (SK)?
2. What are system-level workers’ (immunization directors and managers) perspectives on system-level barriers to and facilitators of HPV vaccination across Saskatchewan?
3. How has the COVID-19 Pandemic disrupted the school-based HPV immunization programme in terms of the scope of the problem posed and the current mandate across Saskatchewan to reach HPV vaccine coverage targets?
4. What do programme documents say about the operationalization of the HPV vaccination system (i.e., programme planning, roll out, and administration of HPV vaccines) across Saskatchewan?

**Figure 1.**
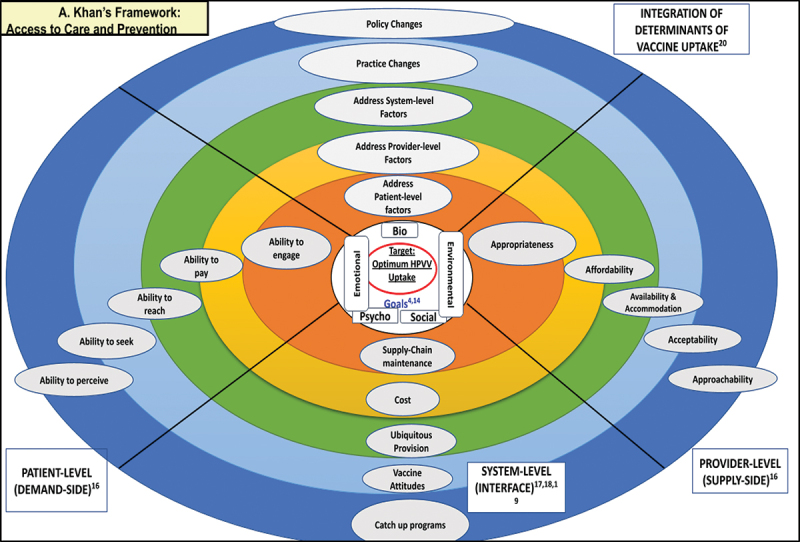
A. Khan’s Framework: Access to Care and Prevention.

Using various data collection methods (interviews and document analysis), we obtained an in-depth understanding of the problem of suboptimal uptake of HPVV by exploring the system and provider-level perspective on the barriers to and facilitators of HPVV. Our review (Khan et al., 2023) on the barriers and facilitators of HPVV uptake across English Canada set the foundation of this research. In our review, lead author A. Khan advanced an analytical framework (see Figure 1) called “A. Khan’s Framework: Access to Care and Prevention” that adapted a patient and provider-level theme (supply-side and demand-side determinants) from Levesque’s conceptual framework of access to health care (Levesque et al., 2013) weaving in the system-level determinants of vaccine uptake (Beshears et al., 2016; Crawshaw et al., 2022; Fisk, 2021) and the determinants of vaccine uptake (Bhugra et al., 2021) for integration. This new combined framework (A. Khan’s Framework: Access to Care and Prevention) serves as our overall study’s theoretical and analytical framework, in which we explore both patients’ and stakeholder perspectives on HPVV uptake. The results of the patient-level analysis are reported elsewhere.

### Study setting

SK has a land area of 577,060.4 square kilometres with a population density of roughly 2.0 people per square kilometre (Government of Canada, Canada S. Focus on Geography Series, 2021Census - Saskatchewan, 2022). With this large geographical space, SK has a population of 1.1 million (Saskatchewan, 2023) approximately half of which is sparsely distributed throughout the province outside the major cities. SK has three major cities: Saskatoon, Regina, and Prince Albert. Saskatoon (266,141) (Government of Canada, Canada, S. Profile table, Census Profile, 2021Census of Population - Regina, City CY [Census subdivision], Saskatchewan;Saskatoon, City CY [Census subdivision], Saskatchewan, 2022) and Regina (226,404) (Census, 2022) have almost similar population sizes, but Prince Albert has much less than half the population of Saskatoon (only about 38,000 people) (Government of Canada, Canada, S. Profile table, Census profile, 2021Census of Population - Prince Albert, City CY [Census subdivision], Saskatchewan, 2022). The two largest cities (Saskatoon and Regina) have populations of roughly 300,000 each, and the rest of the province is made up of small cities such as Prince Albert and rural towns and farming communities, plus the north which has a similar population to Prince Albert but spread over the whole northern half of the province.

The province is under the Saskatchewan Health Authority (SHA) and the Athabasca Health Authority. The southern 2/3 of the province is covered by SHA solely under provincial jurisdiction, while the northern 1/3 is covered by Athabasca health authority governed in a tripartite arrangement between the provincial government, the federal government, and the Athabasca Health Authority (Structural Profile of Public Health in Canada: Indigenous health National Collaborating Centre for Healthy Public Policy, 2019). School locations are more widely spaced out as the population becomes sparser, making school-based immunization programmes more logistically challenging.

Although this study was conducted in Saskatoon, the largest city in the province of SK by population and area (Wikipedia contributors, 2023) the interviews with stakeholders (providers and system-level workers) were conducted from across SK throughout four integrated service areas. In SK, the HPV vaccine is offered through publicly-funded school-based vaccination programmes through coordination between local public health units and school staff to all students in grade six (HPV vaccine access in Canada, 2022, 2022). Besides the school-based programme, the HPV vaccine can be administered at public health clinics to catch up for missed opportunities. Public health nurses collect informed consent letters and administer the vaccines.

### Participants recruitment

We employed a purposive sampling strategy for participant recruitment from across SK throughout SHA. We aimed for diversity in the sample based on the roles and positions of the participants and the area/region they work in for initial interviews and follow-up interviews to obtain a representative sample. We aimed for diversity in the sample to procure rich insights and capture multiple and different perspectives that fulfil the study aims by allowing exploration of barriers to and facilitators of HPVV uptake.

In the context of this study, we classified stakeholders into provider and system-level workers. The provider-level participants include frontline workers and supervisors who work at the provider level in the HPV immunization programme, planning, roll-out/delivery, and administration and represent the population and public health staff from primary health care networks. The system-level participants include directors and managers who work at the system level in the HPV immunization programme, planning, roll-out/delivery, and administration and also represent the population and public health staff from primary health care networks.

In collaboration with XXX and YYY, the study recruitment e-poster was circulated among the population and public health staff from primary health care networks for recruitment for initial interviews. XXX and YYY facilitated the identification of participants geared by a network of people working in vaccination-related roles across all four Integrated Service Areas (ISA) (Saskatoon, Regina, North and Rural) under the XXX. A population health research specialist who is a trained physician recruited the participants by working closely with a Senior Medical Health Officer and Epidemiologist with a vast experience with vaccination-related research. Using the same network, we again reached the same target population for the follow-up interviews.

We reached the potential participants through email with participant recruitment material consisting of an invitation letter, an information sheet briefly describing the study, and a consent form. We scheduled interviews with the participants who expressed interest in contributing to the study. On the recruitment poster and the information material shared, we clarified that the participants would not be compensated for participating in the interviews for this study and that consenting to be interviewed means contributing to a better understanding of the problem in question. Therefore, no participants were provided compensation. Moreover, we conducted the interviews during work hours, and these were deemed relevant to the participants’ work.

### Data collection

We conducted individual semi-structured interviews between October and December 2022 and follow-ups between March and April 2023. Based on participants’ preferences, all interviews were conducted over virtual platforms (either zoom or Webex) in Saskatoon at the XXX at XXX. The office is well-equipped for audio and video conferences and suitable for conducting interviews. In total, we conducted forty-one (*N* = 41) interviews with (*n* = 31) initially and (*n* = 10) follow-up interviews. Based on the stakeholder classification we made, the participant distribution was as follows: In initial interviews, provider-level participants were (*n* = 15), whereas the system-level participants were (*n* = 16). In the follow-up interviews, provider-level participants were (*n* = 6), and system-level participants were (*n* = 4). All the interviews were audio recorded. Initial interviews lasted 1 to 1.5 hours, whereas the follow-up interviews lasted a maximum of one hour.

We used an interview guide (see supplementary material, interview guide 1 and 2) informed by our review and guided by A. Khan’s Framework: Access to Care and Prevention (Khan et al., 2023) for developing interview questions drawing on the most prominent themes related to the HPVV uptake factors in the broader context (English Canada) that emerged from the review (Khan et al., 2023) and identified in the A. Khan’s Framework: Access to Care and Prevention (see Figure 1). During the initial interviews, we collected brief demographic information from the participant to learn about the participants’ characteristics. We asked participants about their perception of the operationalization of the HPV school-based immunization in SK before the COVID-19 pandemic—also the COVID-19-related disruption of the school-based HPVV programmes.

The follow-up interview guide (see supplementary material, interview guide 3) was developed from key themes emerging in the analysis of the first set of interviews. We combined stakeholder groups (system and provider level) and interviewed a subset of the same participants interviewed in the initial interviews. Conducting interviews this way allowed us to expand on the areas of significance that were still ambiguous and find agreements, disagreements, resonances, dissonances, and nuances as a part of data crystallization. We commenced data analysis immediately following the first interview and continued until we obtained thematic saturation (Green & Thorogood, 2018; Saunders et al., 2018).

We also conducted a *document analysis* and reviewed all publicly available provincial documents from January 2015, until July 2023, that included information on HPV immunization. Data were retrieved from the official database of Saskatchewan’s resident health access and immunization services website (Immunization services, 2023) using the keywords HPV immunization, HPV vaccination and HPV vaccine. In addition, we obtained some documents from the immunization leads, specialists and coordinators. We reviewed the following data sources: Saskatchewan Immunization Manual, Vaccine-Preventable Disease Monitoring Report, Guidelines for Administering HPV-9 PPHS—Specialty Immunization and Travel Health Centre, Grade 6 Immunization Package and Provincial School Immunization Policy.

### Data analysis

All data were audio-recorded and transcribed verbatim. Data analysis was facilitated using the qualitative software NVIVO 12. We analysed the two sets of interview data using a thematic analysis approach by adopting hybrid inductive-deductive coding and following the six pragmatic steps described by Braun and Clarke (Braun & Clarke, 2006). The six steps included data familiarization, generating initial codes, searching for themes, reviewing the themes, defining, and naming the themes and producing the report (Rezaei Aghdam et al., 2020). Data for document analysis was extracted by carefully reading the selected documents several times. We analysed the abstracted data from the document review with the interview data using a thematic approach to identify possible barriers to HPVV uptake and potential areas of improvement to enhance HPVV uptake. We organized the data extracted from the selected documents on three axes (target, action and means) of the WHO International Classification of Health Interventions (ICHI) tool (World Health Organization, 2001). We carried out deductive data coding based on the lead author’s HPVV uptake framework (A. Khan’s framework) and inductive coding for emerging themes. Six key themes were identified, ranging from the ubiquitous provision of HPVV and acceptability to vaccine attitudes.

We aimed for *crystallization*, a type of data triangulation, to compare different data sources to enhance the trustworthiness of the study findings by allowing the exploration of diverse perspectives and an opportunity to corroborate and elaborate some of the claims made by the study participants. We aimed not only to find convergences of findings at some specific “truth” but also seek dissonances among different data sources and participants to facilitate our understanding of why (Tracy, 2010) there is a suboptimal uptake of HPVV despite the wide availability of this vaccine through the publicly funded programme. Also, to enhance the trustworthiness of the study findings, we conducted a member-checking exercise by discussing study findings during the follow-up interviews with the participants we interviewed initially. Doing so allowed the participants to comment on the emerging themes, reflect on the study findings and provide suggestions. We also ensured including the voices of multiple participants to capture diversity in perspectives and enhance study rigour.

### Ethical considerations

We sought approval for the study and evaluation protocol (including recruitment strategies and data collection tools) from the University of Saskatchewan’s Behavioural Research Ethics Board (Application ID#: 3545). We also obtained a letter of authorization to conduct research from the Saskatchewan Health Authority. We collected informed consent from all the participants before the interview. Before data analysis, we removed all identifying data in the verbatim transcripts.

## Results

### Participant characteristics from initial and follow-up interviews

We conducted a total of 41 stakeholder interviews. Overall, there was a nearly even split between the type of stakeholders interviewed in phase 1, with 49% provider-level participants and 51% system-level participants. In phase 2, however, the divide was 60% (provider-level participants) and 40% (system-level participants). We interviewed two types of health professionals in both phases: Medical Health Officers (MHOs) and Public Health Nurses (PHNs). The PHNs interviewees reported assuming different working roles and positions that included: immunization coordinators and specialists, PHNs clinical supervisors and consultants, front-line PHNs, directors, executive directors and managers of primary healthcare, and supervisors, managers, and directors from Clinical Integration, Clinical Standard and Professional Practice.

Saskatchewan Health Authority operates through health networks across Saskatchewan through four main areas called Integrated Service Areas (ISA). The four ISA includes Saskatoon, Regina, North, and Rural. In both phases, our participants’ representation was highest in the Rural (*N* = 17) and lowest in Saskatoon (*N* = 3). Participant representation from Regina was in the mid, i.e. (*N* = 6) in the initial, which decreased in follow-up interviews (*N* = 2). In contrast, this was higher in the North in initial interviews (*N* = 11) than in the follow-up interviews (*N* = 2). Please refer to Table I for detailed demographic characteristics of stakeholders participants from initial and follow-up interviews.

**Table I.** Demographic characteristics of stakeholders participants from Initial and follow-up interviews.

Characteristic	Phase 1: n (%)	Phase 2: n (%)	Total: N (%)
Total Number of Stakeholders* Participants	31 (100)	10 (100)	41 (100)
Provider Level Participants	15 (49)	6 (60)	21 (51)
System Level Participants	16 (51)	4 (40)	20 (49)
**Professions, Roles, and Positions**			
Public Health Nurse	7 (22)	2 (20)	9 (21)
Immunization^+^ Coordinator & Specialist	5 (16)	1 (10)	6 (14)
Public Health Nursing Clinical Supervisor & Consultant	5 (16)	1 (10)	6 (14)
Front-Line Public Health Nurse	2 (7)	0 (0)	2 (7)
Medical Health Officer	5 (16)	4 (40)	9 (22)
Director, Executive Director & Manager of Primary Healthcare	5 (16)	0 (0)	5 (12)
Clinical Integration, Clinical Standard and Professional Practice: Supervisors, Managers, Directors.	2 (7)	2 (20)	4 (10)
**Integrated Service Area under Saskatchewan Health Authority**			
Saskatoon	2 (7)	1(10)	3 (8)
Regina	6 (19)	2(20)	8 (20)
North	11 (36)	2(20)	13 (31)
Rural	12 (38)	5(50)	17 (41)

*This category includes workers from both: system as well as provider-level..

^+^Immunization Coordinator includes both: those who are Public Health Nurses (PHNs) and assuming the role of immunization.

coordinator and those who are not PHNs.

### Provider and system level barriers to and facilitators of HPVV uptake

We report barriers to and facilitators of HPVV uptake from interviews and document analysis based on the key themes. The key themes are 1. Information, Awareness and Education. 2. HPV Vaccine-Related Logistics. In discussing the results, we further divide these themes and subthemes related to barriers to or facilitators of HPVV uptake. We found similar key themes in initial and follow-up interviews with system and provider-level participants; however, they vary slightly based on the point of emphasis; for example, provider-level stakeholders thought that the vaccine information sheet was too technical to read and understand, whereas system level stakeholders believed that it is the mode of distribution of the vaccine information sheet (via only paper copies) that serves as the biggest roadblock in ensuring the information reached to parents.

#### Information, awareness and education: barriers

This first key theme and the corresponding sub-themes fall under the following determinants of vaccine uptake included in the A. Khan’s Framework: Access to Care and Prevention (Khan et al., 2023) (see Figure 1): vaccine attitudes, approachability and ubiquitous provision. These are the system and provider-level determinants driven by the psycho-social elements in the A. Khan Framework. Table II provides a detailed overview of stakeholder perception of barriers to and facilitators of HPVV under theme#1 (information, awareness, and education): sub-themes, factor type (barrier vs facilitator), and exemplar quotes.

**Table II.** Stakeholders’ perception of barriers to and facilitators of HPVV under Theme#1 (information, awareness and Education): sub-themes, factor type (Barrier VS Facilitator), and exemplar quote.

A. Khan’s Analytical Framework (Biopsycho- Social Determinants)	A. Khan’s Analytical Framework (Provider and System Level Determinants of Vaccine Uptake)	Main Theme	Sub Themes	Barrier OR Facilitator	Stakeholders Group	Exemplar Quote
Psyco-Social	Vaccine Attitudes	Information, Awareness and Education	Children & Youth Education	Facilitator	Provider-level	*“There is one thing that we used to do years ago, and it kind of went away, and that’s the education piece with the students and in the classroom. As time passed, the public health nurse’s role changed a little within the school. We used to do more, going into the classroom and direct education with students, and I do really feel that that is a loss that we’re not doing that anymore. Lots of vaccines are about trust. Sometimes it doesn’t always work, but if we go in and show our face and say, “This is who I am, I’m not scary. And this is why we’re doing this, and this is why it’s important,” so they understand how vaccines work, and they understand why we’re coming to the school. We’re not just mean nurses coming in to poke them. I think that’s important.” … …* .(Provider level Participant, Interview #2)
			Target Channels of Communication Youth Use	Facilitator	System-level	*“For this, we have to look at most of the channels of communication that the youth use, and that’s mostly social media. I think we need to put some message on social media, Instagram, or most of the other, TikTok, what they are using, a message there.” … …* .(System level Participant, Followup Interview #9)
			Public Health Nurse & Front-Line Staff	Facilitator	Provider-level	*“Personally, I am dedicated to educating the vaccine providers, my frontline nurses, to ensure that they have the appropriate, timely resources to address the needs of those students in the schools. I work with school divisions. I’m going to continue to develop those relationships, to explain to them so that they can support the schools, teachers, and their staff on that end so that those public health nurses and those teachers know that it’s a good program for these students and any education that we can provide to those students is an important piece of the success of any immunization program.” … … …* .(Provider level Participant, Interview #9)
			Restoring Trust through Education on Public Health & Staff	Facilitator	Provider-level	*“I think we need to do better education, of course, whether it’s social media or going to the schools, and I feel the face of the public health nurse needs to be there more. I know we’re busy, but trusting your public health nurse is so big, it really is. It makes me sad when I go to my school, and my kids go to my school, and the kids go, “Ahhh,” because they know needles are coming. I used to go and talk to kids or pick up or whatever; you’re just there; you don’t want to be that scary face. …*.(Provider level Participant, Initial Interview#13)
			Parental Awareness and Education	Facilitator	Provider-level	*“For parents, too, parents need to know who their public health nurse is, who to call and that we are there; we’re still there and there to help. I think that’s important. Education can always improve. Always, always.”…*(Provider level Participant, Interview #4)
			Misconception: HPV is a controversial vaccine	Barrier	Provider-level	*“Some common ones that we see are that some parents think we’re giving it too young, like, “why are we giving it now?” Some feel it might encourage sexual activity, and some are concerned about eh safety of the vaccine.” … …* . (Provider level Participant, Initial Interview#1)
			Misconception: All vaccines but HPV	Barrier	Provider-level	*“There are still a few parents who feel that by immunizing their children for HPV, they are promoting sexual activity. And that they know for a fact that their children are not sexually active. They will not be sexually active until they’re married, and there is no need for this. That is another common belief because they will consent to other vaccines, **not HPV**. Historically speaking, it is similar to how the hepatitis B vaccine was when it first rolled out and then, more people adjusted to that and started accepting the hep B vaccine as well. The HPV has been, I’d say, slightly lower in people changing their mind.”………*.(Provider level Participant, Initial Interview#5)
			Preconception Informed by Religious Belief	Barrier	Provider-level	“*For some, for religious reasons, they think abstinence is the best option, and their children will not be sexually active and won’t come in contact with it. And we run into that a little bit too. I think it sometimes has more to do with the fact of their age and that they see it more as a vaccine that’s for a sexual infection as opposed to causing cancer and why we give it so young. Sometimes it’s just a bit of education with them to explain why we do some things. Sometimes parents opt to receive it; some still choose not to.”* … … .(Provider level Participant, Initial Interview#8)
			Interactive sessions and Education at all societal levels	Facilitator	Provider-level	*“And maybe a way to offer it to parents would be if you educate the teachers and the principals of the school, and then the teachers and the principals could also be the go-between to say, because they’ll know what children probably are not getting any vaccines, including HPV, or even just HPV. And so, if they could say, “You know what,” whether they’re at a teacher conference meeting or the beginning or end of school, or whenever, if a teacher could say to them, “Hey,”- whatever. The teachers have to be careful because they can’t say, “Hey, I know your child’s behind on vaccines.” But if they just said, “Yeah, the public health nurse,” or whomever, “they know a lot of parents have concerns. They can talk to you. You know what, you could talk to them on a Zoom or a phone call or whatever,” and have that face-to-face. I think you have to combat the negative stigma it has. I don’t know how to do that except face-to-face conversations with, like I said, all levels of society, whether it’s a church, early childhood educators, parents, or grandparents. Just the messages of the dangers of HPV and the vaccine’s safety.”……*.(Provider level Participant, Initial Interview#6)
			Misconception: HPVV, a sex-related vaccine	Barrier	System-level	*“Yes, initially, we did find some religious objections to it because that was probably their misconception or misunderstanding that, in a way, this vaccine would encourage children to get involved in risky sexual behaviour. Some groups thought that giving HPV vaccines is like providing them with a free pass to involve in sexual activity.” … …* .(System level Participant, Initial Interview#1)
			HPV-related gender predilection	Barrier	System-level	*“Yeah, and when it came out, the focus was on the cervix, right? And what with one vaccine? The one GSK vaccine was even called Cervarix, and it only had 16 and 18 in the component, so it does a bivalent vaccine. So, there is a lot of focus on the cervix, which is just not the right spot for some parents, but now that we have both genders getting it. We talk about head and neck cancers and the increase of those things, and we are trying to prevent all of these cancers, and that should be the story, not, you know, who’s going to have sex.”……*(System level Participant, Initial Interview#14)
			Morally Objectionable	Barrier	System-level	*“I do think the moral objection concept comes into play in this situation as well, where maybe some parents don’t want to believe their children are approaching that age, or maybe are engaged in sexual activity and either don’t want to believe it or are uncomfortable with that conversation and aren’t sure how to approach it or just don’t want to approach it. So, I think that is also a big part of it.”* (System level Participant, Initial Interview#8)
			Target different groups (parents, families, children and youth) to spread Awareness, Provide info and handle Misinformation.	Facilitator	System-level	*“I’d really like to see reinvestment again and comprehensive school. Basically, entrenching health more intensively within the school curriculum. You know the immunology vaccinology is a component of their biology courses, and so it ties in nicely. In essence, those kids are getting much more valuable informed consent versus the others, and so that’s what I’d really like to see is that’s just a great example but the whole sweeping concept of taking a look at the curriculum to help strengthen health literacy and line it in with some of these very predictable patterns. We know when to start offering comprehensive sexual education based on biological development during the kids; how do you build that in and again normalize some of that work? And the one we start offering things we know when they’re going to be coming into schools and certainty that the curriculum can align a bit to help build some knowledge and expertise so that they can have that valuable conversation with their families.”……*.(System level Participant, Initial Interview#11)
			Target different groups (public health nurses and immunization staff) to a periodic Education	Facilitator	System-level	*“And being able to address that and being able to say — as a public health nurse, I think we need to provide the education. We have so much churn right now that I would say that it takes a while. It takes that experienced public health nurse, often not 100%, to really feel confident and so — I don’t mean challenge the parent, but not be afraid of the parents’ questions or if the parent’s like, “Oh, no, I don’t want them to have HPV,” to be able to say to that parent, “Oh, okay, so Hepatitis B is good, so can I ask why you’re not interested in HPV for your child?” Because then that way I know what to address, like what are your questions around it?”……………*(System level Participant, Initial Interview#15)
			Vaccine Info Sheet: Amend to address people’s concerns	Facilitator	System-level	*Now that I’ve heard, there might be concerns about, you know, the information sheet. If that’s going to contribute to people not providing consent, then definitely those concerns will have to be addressed, and some of them can be addressed in the info sheet. Still, I also think there are specific issues like infertility, right? That’s usually addressed in Q&A, just like we had to do with COVID, right? There were many misconceptions about COVID vaccine, and we addressed those in Q&A documents because you don’t want to leave people in the dark. You want to respond to them if you know the concerns and find the appropriate place to address them, right?”…*(System level Participant, Initial Interview#13)
Environmental/ Contextual	Approachability		Target different groups (community health workers) to Education via community outreach	Facilitator	System-level	“*I sometimes think we have community health workers in some of our programs. It would be great if we somehow had something in our networks, like a community health worker, particularly for school-age like grade six, where they can reach out to the parents; they may have more capacity to answer parents’ questions. — Maybe if I have a school that has a high rate of, let’s say Ukraine or a high rate of people from Syria, or whatever, that you would have a community health worker with that background that can connect with families and help them with not only our process in Saskatchewan but with any questions they have. Giving them training about vaccine hesitancy. Giving them back the ability to connect with people with a similar background where they feel maybe more comfortable talking to that individual about HPV.”…*(System level Participant, Initial Interview#4)
	Ubiquitous Provision		Physicians work as a part of the team	Facilitator	Provider-level	*“All health care workers. Every opportunity, physicians, any opportunity. If they know that the children are not vaccinated or need vaccines, education at all times. Answer their questions. In every population, every person trusts a different person, a different healthcare worker. If a public health nurse says it, sometimes they just won’t listen, but if they go to their doctor and the doctor says it, then they’ll listen. Not necessarily the giving of the vaccines, just the education piece. I really think that’s important, especially with vaccines like HPV. I’ve said numerous times trust is big. We really need to work together; we need to be one big team. We all have to be on the same page and understand the science behind vaccines and that they’re important, this is why it’s safe, and this is why we should do it. There are so many different access points.”……*.(Provider level Participant, Initial Interview#2)

Provider-level stakeholders communicated that one of the many barriers is inadequate awareness or low literacy on HPV infection and HPVV. They emphasized educating three key population subgroups: youth, parents, and school staff. They also highlighted a need for clear messaging in the school and for providing additional educational avenues for children and youth because they are the ones who get needles in their arms. Provider-level stakeholders articulated that children and youth education about HPV infection and HPVV is critical. They believed investing in students’ education would help establish trust in the vaccine and providers.

Similarly, system-level stakeholders reported insufficient avenues for information exchange with parents. They highlighted that parental awareness of existing avenues is important to enabling them to navigate the information package sent to them through the school-based immunization programme. They highlighted a need for parental education in multiple dimensions, including providing the right information, dismantling myths, and misconceptions, and addressing specific questions and concerns by adopting a person-centred approach. They exemplified that parents recognize HPV as a sex-related vaccine.

One is parents’ preconception that HPV is a sex vaccine; it might give young ones the freedom to involve themselves in risky behaviours.” … … …. (System level Participant, Initial Interview#6)

Provider-level stakeholders advocated for periodic training and refresher courses on HPV infection and immunization for health care staff, including immunization staff and public health nurses. They urged that engaging school staff through educational workshops would help them cope with the students who do not bring the consent form back to school and may motivate them to bring back consent. They also stressed targeted education to vulnerable communities and discouraged reluctance and hesitancy in reaching out and educating these population subgroups due to the fear of singling them out.

So, I think targeted education is the key. We have to take a closer look to see if there are sub-populations; if we can identify that, then targeted education to those populations would be helpful, but I understand we struggle with that because nobody wants to single anyone out and go out specific populations. The education piece is important that targets sub-populations who have maybe caught onto the misinformation that’s circulating, and this should be done with a consistent approach all across. … … … … .(Provider level Participant, Initial Interview#11)

In addition to highlighting the population subgroups that should be targeted for educating about HPV infection and HPVV, HPVV-related misconceptions must be addressed. In that, they cited that parents consider HPVV a controversial vaccine. They also reported observing a misconception among parents about using the HPVV and the potential of promiscuity in youth, which served as an important barrier to HPVV uptake. Due to similar reasons, many parents were not convinced that the HPVV should be given to children, as they do not believe their child(ren) is at risk. Moreover, parents were reluctant about the HPVV due to their concern and uncertainty about HPVV safety.

System-level stakeholders emphasized that the most prevailing misconceptions about HPVV among parents were driven by their fear that getting HPVV would make their children promiscuous. These misconceptions support parents’ belief that their child(ren) will not be involved in sexual activity and will completely abstain from it until marriage; therefore, they do not require the HPVV. Additionally, system-level participants cited that parents face several barriers to seeking HPVV: being too young to get the vaccine, the belief that their children are not sexually active, the risk of encouraging them to involve in sexual activity or related risky behaviours, and that HPVV is for females only as it can protect them from having cervical cancers.

System-level stakeholders also expressed concerns that parents were uncomfortable conversing with their children about the HPVV and the why of its importance because, as some parents find the administration of the HPVV morally objectionable, others are not yet ready to believe that their children will be reaching the age where they will be sexually active, and this makes it more difficult for parents to navigate the situation then and decide to consent or decline the HPVV.

System-level stakeholders identified inadequate physicians’ leadership outside public health to help enhance HPVV uptake. They highlighted that people seek healthcare in many ways and that there are insufficient avenues that could remind people about HPV infection and highlight the role of HPVV in preventing HPV-linked cancers. They believed that periodic education with a repeated offer of HPVV at different healthcare encounters would collectively work to serve the purpose.

But it’s another space where we don’t have the best at all, is that, okay, if we decide that we can’t build capacity like we want, amongst pharmacists, amongst NPs, amongst primary care physicians, amongst massage therapists—any other care providers, people seek healthcare in a lot of ways—there is very little to no mechanism for them to refer in a way that matters for the client, to a public health nurse. To help facilitate a conversation. If they want to refer to public health, it’s not tidy. It’s actually really messy. We don’t have a place to send. We don’t have a vaccine-hesitant clinic.… … … .(System level Participant, Initial Interview#8)

#### Information, awareness and education: facilitators

System-level stakeholders identified important groups that need education about HPV infection and the role of the HPVV. They strongly suggested educating parents, families, children, and youth by incorporating health literacy in the school curriculum, expanding the concept of immunization and providing vaccine education adopting a health-promoting school model. Educating the vaccine receivers (children) would be valuable as the vaccine end-users will converse with their parents and families serving as vaccine consenters. Moreover, this idea reinforces valuable informed consent.

The idea of using health promoting schools model, which we do struggle a lot to get in Saskatchewan, would be a big facilitator, because, by default, everyone who attends school on a regular basis at that level is certainly a space for them to understand and start exploring, and be able to know how they can take steps if they want to know more and do want to seek immunization.*……*.(System level Participant, Followup Interview#9)

Additionally, provider-level stakeholders emphasized targeting the challenges of communication youth use these days to put a message about HPV infection and the role of HPVV. Besides, PHNs and other immunization staff should be offered periodic education through workshops, refresher courses or training in a persistent fashion. Doing so would equip the staff with the knowledge and confidence required to handle the stigma attached to HPVV and address parental concerns better. This may range from engaging the parents in conversations and managing the components of vaccine hesitancy in them at large.

Provider-level stakeholders identified school staff amongst the important groups instrumental in navigating immunization administration between PHNs and parents of the child(ren) behind in immunization without confidentiality breach. Therefore, it is crucial to first engage with school staff, highlight the importance of HPVV, and collectively work on a plan to connect with parents so that PHNs can address parents’ concerns in case-by-case consultations to avoid the stigma attached to the HPV vaccine.

Provider-level stakeholders cautioned that teachers should carefully set this connection up to avoid confidentiality threats and any change to enhance stigma for those behind in immunization. One-on-one interaction opportunities between PHNs and parents and group interaction with school staff were considered important avenues that could facilitate HPVV uptake. In addition, educational campaigns, the use of social media, and infographic posters in school were additional pieces stakeholders believed would help improve vaccine confidence and increase HPVV uptake.

I think we need to do better education, of course, whether it’s social media or going to the schools, and I feel the face of the public health nurse needs to be there more. I know we’re busy, but trusting your public health nurse is so big, it really is. It makes me sad when I go to my school, and my kids go to my school, and the kids go, ‘Ahhh,’ because they know needles are coming. I used to go and talk to kids or pick up or whatever; you’re just there; you don’t want to be that scary face.*……*.(Provider level Participant, Initial Interview#13)

Other groups that the system level-stakeholders identified require education included Community Health Workers (CHW) and religious leaders and stakeholders. Stakeholders highlighted that educating the CHW would serve as impactful facilitators through their unique role in community outreach, especially in reaching population subgroups and addressing their concerns. CHW’s training should also involve learning ways to deal with different subgroups in the community. Stakeholders highlighted that it would be ideal to have CHWs with diverse backgrounds who can connect well with diverse communities across SK.

System-level stakeholders highlighted the importance of educating religious leaders and stakeholders as a good section of society follows them and relies on them in decision-making. Therefore, providing religious leaders and stakeholders with health information in a broader context and sensitizing them about the HPVV would be an excellent way to create awareness. In this way, an avenue can be identified and set up to address people’s concerns about taking certain kinds of medicine because they think taking a vaccine is prohibited by religion in some sense.

Some system-level stakeholders also advocated for physician leadership outside of public health to promote HPV immunization by educating people directly, engaging with them at the first opportunity, and trying to use different points of healthcare-related access to remind them of HPVV and the consequences of long-term HPV infection.

In every population, every person trusts a different person, a different healthcare worker. If a public health nurse says it, sometimes they just won’t listen, but if they go to their doctor and the doctor says it, they’ll listen. Not necessarily the giving of the vaccines, just the education piece. I really think that’s important, especially with vaccines like HPV. I’ve said numerous times trust is big. We really need to work together; we need to be one big team. We all have to be on the same page and understand the science behind vaccines, and that they’re important, this is why it’s safe and why we should do it. There are so many different access points.*…………*.(System level Participant, Initial Interview#2)

#### HPV vaccine-related logistics: barriers

This second key theme and the corresponding sub-themes fall under the following determinants of vaccine uptake included in the A. Khan’s Framework: Access to Care and Prevention (Khan et al., 2023) (see Figure 1): availability and accommodation, approachability, appropriateness, acceptability, and cost. These are the system and provider-level determinants driven by the environmental elements. Table III provides a detailed overview of stakeholders’ perceptions of barriers to and facilitators of HPVV under theme#2 (vaccine-related logistics): sub-themes, factor type (barrier vs facilitator), and exemplar quotes.

**Table III.** Stakeholders’ perception of barriers to and facilitators of HPVV under Theme#2 (vaccine-related logistics): sub-themes, factor type (Barrier VS Facilitator), and exemplar quote.

A. Khan’s Analytical Framework (Biopsychosocial Determinants)	A. Khan’s Analytical Framework (Provider and System Level Determinants of Vaccine Uptake)	Main Theme	Sub Themes	Barrier OR Facilitator	Stakeholders Group	Exemplar Quote
Environmental/ Contextual	Availability and Accommodation	Vaccine- Related Logistics	Vaccine Info Sheet: English as a Second Language	Barrier	Provider-level	*“I think people are assuming English as a second language, that they don’t get done, but I find that they are the first ones if we have the information like if we have their immunization history, they’re the first ones to sign their consents and bring them back. And they’re agreeable to getting COVID and flu vaccines when they get their regular infant vaccines when they’re eligible.” … …* (Provider level Participant, Initial Interview#10)
			Vaccine Info Sheet: English as a Second Language	Facilitator	Provider-level	*The vaccine information sheet, in general, is probably at a great succeeding level. The only thing that might basically is with an increasing number of immigrants, we may need to have the vaccine sheet translated into some other languages that are out there. I know, for example, we had a number of Ukrainian immigrants. And now, there will be a large number again. I would have to get translated before we could get a new form of consent.”………*(Provider level Participant, Initial Interview#11).
			School spacing environment and the Confidentiality issues	Barrier	Provider-level	*“Sometimes, the schools are struggling for space; they don’t have extra space. There are kids in every corner of the school, so when we get there, sometimes we don’t have a regular space that we use all the time in most schools; we’re kind of just put in a different space every time, we’re working in small rooms or almost like storage closets where we have to set up and maybe don’t have the proper table and enough chairs. When people are getting vaccinated, especially because they do it in from of the whole school, they used to be that they didn’t take them and put them in a different room and vaccinated them. They would just do it in the gym. We’d line them up and just go down the row. I think one of the barriers to that was if you were getting two injections, people knew what you were getting. There was some stigma attached to that, like, “Oh, she’s getting the HPV vaccine. She’s going to be able to go have sex …* .(Provider level Participant, Initial Interview#14)
			Under Staffing: A Constant Challenge	Barrier	System-level	*“We complain about short staffed and then just keep working, and that’s our worst enemy because the people sitting at the top think, “they don’t have any problems; they can work.” As I said, in the last 20 years, I have seen in my area that public health has been ripped off in every opportunity of staff. Whoever resides in public health, that position is abolished. We are bare bones in public health, and we are still keeping going on. I think the ministry has to be able to see this. An analysis or inventory of public health nurses based on the population. In Saskatchewan, the population is less, but the area is so large; if I’m not wrong, the ministry suggests one public health nurse for a 3,500 population. If you look at this, this is not the case in most of the rural and modern Saskatchewan population”………*.(System level Participant, Initial Interview#10)
	Approachability		Vaccine Info Sheet: Hybrid Mode of distributing immunization packages	Facilitator	Provider-level	*“Well, my thoughts, probably if you could do a blended method. So, you could do paper or electronic, whatever a parent wanted. Because some parents are really techy, and they know things are coming into their inbox, they’ll be checking and seeing it. “Great, yeah, let’s do that,” get it back, dat-dat-dat, get it back, good. Versus other parents don’t have the capability to be techy, so then they need that paper to come home, and they need to sign it, and they need to get it back. But of course, that means the child has to get it home, and the child has to get it back. But I mean, if it works for that family, great. So, probably a blended method, depending on what the parent, what works best for the parent, that’s probably what I would suggest.”………*(Provider level Participant, Initial Interview#2)
			Staffing constraints: difficult Catchup for Missed Opportunities, Work overload and Burnout	Barrier	Provider-level	*“The biggest thing’s being missed are we’re short staffed, and when you’re short staffed, you just don’t have the time to do everything as well and as completely as you would. In the past, more time would probably be spent going through our class list. Making sure that everything is more thoroughly up-to-date and more timely. We bring everyone who’s highly delinquent in getting them up to date for any missed opportunities. With our limited resources, sometimes they may have to wait an extra month or two, and we just catch them up when we’re going in to do the school program when we have a number of nurses. It has to do with human resources or public health nurses, and then there could be a somewhat longer delay in catching up with students because we can’t get to them as fast as we would like. From a health side of it, one of our biggest limitations again is human resources. We’re all running very short-staffed. We’re heavily fatigued and highly stressed, having survived COVID. Our sickness rate is through the roof. And doing the schoolwork, making those phone calls, getting the consent, and everything is all limited by not enough public health nurses to do all of the work.”……*.(Provider level Participant, Initial Interview#15)
	Appropriateness		Suboptimal Needle Spacing: Multiple Needles For Grade Sixers	Barrier	Provider-level	*“I feel that some of these grade six students are being traumatized by having to get four vaccines at once, and we are seeing more students wanting to be immunized in our office. Not in the school because of feeling traumatized, and that’s when you may see that with the first child, or we see that with the second round, and that again has reduced our capacity because that’s an extra time. To immunize these kids in school. Some of them want their parents there. I understand. I understand where the students are coming from because I do think we’ve traumatized some. We have asked that we could maybe split up some of the vaccines. I think when the grade eights are immunized there and given one vaccine, and we have discussed possibly with the ministry that they offer them Menveo, that’s the meningitis vaccine. We immunize with four types of meningitis, and that is one dose. We suggested prior to COVID that it’d be given with the grade eight injection, so they would get two at that point and only get two or three in grade six. I see that as possibly being an improvement. And getting HPV uptake improved because some parents do not provide consent because their kids do not want their other vaccines. So the spacing between the vaccines should be six months. Well, it wasn’t, so it’s not optimal spacing.”…*.(Provider level Participant, Initial Interview#9)
			Vaccine Info Sheet: Too Wordy & Technical	Barrier	Provider-level	“*I just compare that to my own family. My husband’s a farmer. If I handed him my children’s vaccine information sheet, he’d take one glance at it and see how much reading he has to do. He’s a Grade 12 graduate. He’s not a college graduate. He does well with his work but can’t be bothered to read. It’s like, just give me the highlights here. The general public just wants the highlights, so sometimes they’re signing these consents and probably not reading the information sheet. One of the things we already talked about was the consent form and the information being too busy and too difficult to read and complete.” … …* .(Provider level Participant, Initial Interview#7)
			Vaccine Info Sheet: Promote HPV Vaccine-Related Cancer Prevention	Barrier		*“I think number one, it’s on the information sheet, one of the first couple lines, “it protects against sexually transmitted diseases,” I think that just stops parents. Because, in their mind, my grade six isn’t sexually active. When I finally get to talk with parents and say, “that’s why we do it so early because they’re not sexually active.” Sometimes, they’re a little bit more understanding. I don’t know why; I think that’s what it is. I don’t know, that’s my personal opinion; when they read the information sheets, they see it’s “protect against sexually transmitted disease,” and they’re only in grade six. There’s a barrier there. Maybe that first because once you start mentioning the prevention of cancer, people listen, or parents seem to listen a little bit more because a lot of them didn’t have that opportunity to have this vaccine. And what vaccine is out there that can actually prevent cancer?” … …* .(Provider level Participant, Initial Interview#12)
			Lack of Appreciation and Early Retirement	Barrier	Provider-level	*“There are two things. It is, yes, of course, funding. To pay the salaries and more money, but then, the other step is just the resource. I’m going to say everyone above me, in general, is not treating healthcare personnel very well, and you have many, many people leaving the healthcare field because of this. Finding the people to put them into the positions will also be a struggle because we do not feel appreciated or valued, and it is a huge factor in the welfare, availability, and functioning of healthcare workers as we see them today.”*(Provider level Participant, Initial Interview#6)
			Vaccine Info Sheet: Mode of distributing immunization packages & Privacy Issues	Barrier	System-level	“*Yeah, I think when you’re looking at improvement, you need to look at barriers. And for me, the big thing that applies to all vaccines we provide schools is definitely the consent process. We need to think about how we can make it easier for parents; how can we make sure that parents receive that request and they send it back? I agree with some parents that hard copies are going to work, but for other parents, electronic copies are going to work, but then also the question about where the electronic copy comes from SHA, schools directly, you know, the whole privacy issue, so consent is at the top of the list. … …*(System level Participant, Initial Interview#2)
	Acceptability		Linkage of School Data System and Panorama	Facilitator	System-level	*“Yeah, and that’s where I, my sweeping statements from earlier of the assigned PHN, reach out to the schools, which they call; I have no idea. Who’s their connection at the school? I don’t know. How they go through that conversation of exchanging information, I don’t know. We’re working towards a process where there should be a push directly from the school data systems to Panorama. That’s really what we want. We want to ensure that because, quite often, what the schools have registered is quite different from what the actual school looks like when you immunize. So, although we’ll send a list, the school’s okay school started; these are the kids we have registered in grades six and eight, and it’s different by the time they go in. There are a lot of kids who have moved in or moved out, which makes the day quite delayed because there are surprises. And so, to be able to get, yeah, so that one was always a bit of a challenge.”…*(System level Participant, Initial Interview#12)
			Staffing Issues: A Political Barrier	Barrier	Provider-level	*“Ideally, it would be great to think that opportunity is going to present. If you want to know how we feel in reality, it’s the exact opposite. We feel that there is an ongoing plan to devolve public health in general. We feel that they are trying to get rid of public health. We function in the long term. The vaccine you see a benefit of it in twenty years. All of your politicians are full in power, running the money; their terms are four years. They want to be able to promote what they have managed to accomplish in acute care because they can see immediate results. How we feel as public health staff is that they are attempting to get rid of us. They are promoting physicians and pharmacists to give more and more vaccines. I have no problem stating factually that we do a far superior job of vaccines. Whole chain administration, documentation, you name it. We do so much better at it as compared to pharmacists and physicians. That is what we do. They do it off the side of their desks, and you can tell. You could probably also tell I’m a little angry about this piece.” …* .(Provider level Participant, Initial Interview#12)
			HPV vaccine to a single dose	Facilitator	System-level	*“I’m really excited about the idea of a **single dose**. I think that’s even in the Hep-B stages too, and part of the reason is then you have two opportunities, right? So, if kiddo weren’t around for the first visit, they’d be behind on their B, but they’ll be able to catch them up on their second dose, I mean their full dose of HPV. So, they only go into the schools once, which means if your kid happens to be sick that day, truant, or have whatever reason that they’re not present, the only opportunity for them to get back on track is through their family who would have to seek that service through a clinic, which then means it’s client-driven instead of being a push to the client.” “Now, the school kids will get **two doses**, right? If they’re outside of that age range, they will need three doses; if they are immunocompromised, they’ll need three doses. But in the future, we know now that one dose is as effective as three doses to prevent all those warts and cancer. We haven’t started to do that yet, but we are waiting for the NACI statement. At some point, NACI will put out their statement saying we can likely offer one dose as the equivalent of three doses. That will take time, maybe a couple of months or six months. At some point, that will happen because there are so many good studies and WHO also recommends only one dose of HPV vaccination for feasibility, and equal protection, compared to three doses. Then, why not one dose moving forwards in the future? Now, that’s just for information. The status is still the same, but at some point, that will happen.”…*(System level Participant, Initial Interview#8)
	Cost		HPV Vaccine Cost: An issue	Barrier	System-level	*“Right, and I know the cost came up; I just remember hearing that from a parent. Her child was a; I can’t remember the exact dates, but there was a cutoff date where it was free, but they were born before that, then they had to pay, and their parent was willing to pay but wanted their child just to be forthcoming of when they were reading to need it. That was a bit of an awkward conversation because it came down to, we know we need it, but we also have to pay for it, so we have billed them before then.”………*(System level Participant, Initial Interview#15)
			HPV Vaccine Cost: Age Cap for HPVV Eligibility	Facilitator	System-level	*“Yet, I do think we would be able to justify promoting the HPV vaccine a lot longer and include other age groups and other risk factors. Right, now, in Saskatchewan, we have an age limit to get the HPV vaccine for free, but what if you’re immuno-compromised, which I think is terrific, regardless of your birth year, that is from nine to 26 still though. It does capture all people. That’s really good, but we **don’t** cover men who have sex with men. We **only** cover males if they’re healthy and born in 2006 or later. But again, I think we also need to target our men who have sex with men as a high-risk category. In any case, it would be great if it was like Hep-B, where **once you qualify, you always qualify**.”…*(System level Participant, Followup Interview#10)

Provider-level stakeholders cited several concerns about the immunization packages sent home via school-based vaccine programmes. These packages contain a vaccine info sheet as well as a consent form. They highlighted that the vaccine information sheet needs to be more concise and less technical. Stakeholders suggested that the vaccine information sheet could be less text-heavy, avoid using many medical terms and serve as a more user-friendly guide, so the lay public can easily read and understand.

Provider-level stakeholders discussed that many people in SK assume English as a second language; therefore, they do not read the information sheet or do not understand it entirely if they choose to read it. As a result, many do not provide consent for HPVV. Stakeholders stressed the importance of understanding the immunization material to guide decision-making for filling out the consent form. Otherwise, the consent obtained might not be “informed consent,” and—is something Stakeholders were not comfortable with hence flagged it as an area that requires further exploration.

I just compare that to my family. My husband’s a farmer. If I handed him my children’s vaccine information sheet, he’d take one glance at it and see how much reading he has to do. He’s a Grade 12 graduate. He’s not a college graduate. He does well with his work but can’t be bothered to read. It’s like, just give me the highlights here. The general public just wants the highlights, so sometimes they’re signing these consents and probably not reading the information sheet. One of the things we already talked about was the consent form and the information being too busy and too difficult to read and complete. *…* .(Provider level Participant, Initial Interview#11)

System-level stakeholders highlighted that the vaccine information sheet is not of substantial standard, and it is designed to be very politically correct or legally precise, where one is given a lot of information with little to no concern if the message it aimed to deliver did get across. Stakeholders believed the vaccine information sheet was the main barrier to HPVV uptake for many reasons, i.e., not up-to-date, had a high reading level, technical, text-heavy and not a user-friendly guide.

Yeah, I think I’ve said the same thing that our sheets are garbage. I actually really like the idea of the language of politically correct, because my mentality about the fact sheets has been due to who produces them and who owns them; there is a significant sense of protection against liability, not about- That’s the priority. Think about the planning [inaudible, 00:29:53]. These sheets, it’s akin to that big, long document inside the pill box, that because they made that, if you have one of those adverse events and you didn’t read it, well, it’s on you now. Right? And that’s the way the fact sheets are right now. They scream I have to put everything, and I have to be very correct about it; I have to be legal. Not politically correct, I would say legally precise, to protect against liability. Which, we certainly know, does not align well; hilariously, what a logical person would dictate is what is necessary to make an informed decision.*…* .(System level Participant, Followup Interview#7)

Stakeholders cited understaffing, time constraints, difficult catchup for missed opportunities, work overload, burnout, lack of appreciation, and early retirements as the most significant and interrelated factors that impede HPVV uptake. They expressed frustration with staffing constraints which leads to difficult catchup for missed opportunities for a variety of child(ren), i.e., truant, sick on needle day, frequent movers, homeschoolers, online learners, and new admission. They also discussed that multiple attempts to reach parents to seek consent for HPVV and difficult catchup with those children who are behind in immunization is time taking and pose challenges to already constrained time. These factors lead to work overload and burnout, which is especially challenging after the COVID-19 pandemic.

System-level stakeholders viewed understaffing as a political barrier as they voiced concerns about not hiring staff in the face of persistent short staffing. They also suspected that such an attitude might be grounded in personal or political benefits geared by the plan to devolve public health. They cited that a lack of recognition of the PHNs’ work and struggle with navigating through the “thick and thin” that comes their way in getting the needle into people’s arms is one of the main factors many staff decide to retire early.

They also recognized that early retirements of the immunization workforce add to the already exhausted staff, time, and their energies hence serving as significant barriers to HPVV uptake. System-level stakeholders counted all these factors as barriers to effective HPVV uptake by end users. They felt that healthcare personnel should be treated well by all despite an existing hierarchy in the system, as a failure to do so poses challenges to the sustainability of the healthcare system hence increasing the immunization workforce.

Provider-level stakeholders also reported some stand-alone barriers in addition to the interrelated barriers discussed above. They reported school spacing for needle administration and confidentiality issues to impact HPVV uptake. They complained that the space allocated by the school to set up a vaccine clinic has less room with no proper furniture. PHNs find it extremely challenging to set up a vaccine clinic in a congested space and be able to administer vaccines comfortably. They were frustrated that schools sometimes do not contribute adequately to help maintain the confidentiality of students receiving needles, especially when PHNs are given gym areas to queue up children and administer the needle to grade sixers. HPVV comes with a stigma, and confidentiality issues could significantly impede HPVV uptake.

Provider-level stakeholders also identified the suboptimal needle spacing for grade sixers as they are offered multiple vaccines in the same grade. They articulated that the grade sixers voiced being traumatized by having many needles at a time due to a suboptimal time between different needle administrations. They highlighted that suboptimal needle spacing was a constant barrier as there had been an effort to change a policy and possibly provide needle spacing between a few vaccines given in series, but the efforts remained unsuccessful. Finally, system-level participants cited HPVV cost as an important issue because they believe getting HPVV is difficult for those individuals who must pay for it once they cross the eligibility age.

#### HPV vaccine-related logistics: facilitators

Provider-level stakeholders suggested changing the distribution mode of immunization material from a paper copy mono-strategy to a hybrid strategy. A hybrid strategy could involve a paper copy with an electronic reminder because solely relying on a child to take the consent form home and bring it back to school has not been the best way to distribute immunization packages as it comes with many challenges, e.g., loss of consent form, etc. Therefore, adopting a hybrid approach would likely enhance the return rate of the consent form because it will cover both types of parent groups those who are more technology smart and those who find paper forms easy to fill and return. They also advocated translating immunization material (vaccine info sheet and consent form) into different languages and making them available through school-based immunization programmes.

Well, my thoughts, probably if you could do a blended method. So, you could do paper or electronic, whatever a parent wanted. Because some parents are really techy, and they know things are coming into their inbox, they’ll be checking and seeing it. ‘Great, yeah, let’s do that,’ get it back, dat-dat-dat, get it back, good. Versus other parents don’t have the capability to be techy, so then they need that paper to come home, and they need to sign it, and they need to get it back. But of course, that means the child has to get it home, and the child has to get it back. But I mean, if it works for that family, great. So, probably a blended method, depending on what the parent, what works best for the parent, that’s probably what I would suggest. (Provider level Participant, Initial Interview#2).

Provider-level stakeholders suggested following the COVID-19 vaccine model to amend the vaccine information sheet and creating a user-friendly guide that contains information on the possible questions, people would like answers to and includes all relevant information. They emphasized the proper promotion of HPVV with a cancer prevention focus instead of a sexual infection focus because a disproportionate emphasis may mislead parents who are already ambiguous about the need for HPVV for young folks who are not yet sexually active. Therefore, reorienting the emphasis by highlighting that this vaccine prevents cancers of cervical, anogenital and oropharyngeal cancers as opposed to starting the information sheet with a focus on sexually transmitted infections. Provider-level stakeholders clarified that reorienting the focus does not mean removing information about STIs but rather rewording appropriately down the information sheet.

I think number one, it’s on the information sheet, one of the first couple lines, ‘it protects against sexually transmitted diseases,’ I think that just stops parents. Because, in their mind, my grade six isn’t sexually active*……*(Provider level Participant, Initial Interview#5)

System-level stakeholders suggested developing a data platform to link the school data system and immunization database where there is a direct flow of information exchange between the two while maintaining confidentiality. In this way, many children who are behind in immunizations can be reached. In addition, stakeholders considered that changing the HPVV from a dosed series to a single shot would enhance HPVV uptake rates. They also suggested removing the age cap for getting HPVV for free or expanding the eligibility age from 27 to 47. Doing so will include a broader range of population subgroups and enhance HPVV uptake rates. Finally, stakeholders strongly believed that a team approach for HPVV delivery and administration is required to settle the spacing issue in schools.

*Document analysis* revealed a few key themes and subthemes that fall under the following domains on A. Khan’s Framework for Access to Care and Prevention: vaccine attitudes, ubiquitous provision, catchup programmes, approachability, availability, acceptability, appropriateness, and accommodation driven by the psycho-social, environmental/contextual circumstances. In terms of facilitators that existed already: documents reflected a broad focus on all population types, including special populations such as those having specific diseases/conditions as well as those whose immunization record is not certain (internationally adopted children from orphanages, refugee children and immigrants’ children) regardless of their place of birth and aimed to get the needle in their arm at the first opportunity. If such practice continues, it will serve as a strong facilitator that keeps HPVV uptake rates high.

Among the barriers noted, several important ones included: First, there was no agreed-upon provincial surveillance case definition for HPV cases, as HPV cases are not reportable in SK. This serious barrier hinders HPVV uptake rates as we do not have statistics to know how many cases we have in SK with HPV-related diseases. Second, guidelines around HPVV effectiveness, safety, and recommended intervals have been given briefly, with appropriate links to the relevant sites for detailed information.

Also, the highlights in the vaccine guideline did not include all the information needed to use HPVV safely and effectively when a client takes the vaccine off-label leaving a space for ambiguity. In that, the highlights could be elaborative and direct. Third, the documents serve as a classic example to illustrate a layperson’s struggle to complete the consent form and clearly understand the role of HPVV due to insufficient information on HPV infection and HPVV. The information provided in the document was not easy to understand, especially for subgroups assuming English as a second language.

### The COVID-19 pandemic impact

We report the COVID-19 pandemic impact based on (1) the problem posed due to the COVID-19 pandemic-related disruptions of school-based immunization, (2) the current mandate to reach the coverage target and (3) lessons learnt. We found two broad factors of prime significance under the impact of the COVID-19 pandemic (a) resource sacristy and (b) vaccine scepticism and reported them based on the type of impact it has posed, i.e., negative or positive. Negative impact reflects factors that impede the uptake of HPVV, whereas positive impact reflects factors that likely enhance the HPVV uptake. Please refer to Table IV for a summary of the COVID-19 Pandemic impact with themes, subthemes and exemplar quotes.

**Table IV.** COVID-19 pandemic impact on school-based HPV immunization programme: categories, themes and exemplar quote.

The COVID-19 Pandemic Impact on School-Based HPV Immunization Program			
Key Themes	Sub-themes	Type of Impact	Exemplar Quote
Resource Scarcity	More staff, time, resources, and organization/planning required	Negative	*“From a health side of it, one of our biggest limitations again is human resources. We’re all running very short-staffed. We’re heavily fatigued and highly stressed having survived COVID. Our sickness rate is through the roof, and we’ve had many job vacancies. Just trying to get into the schools on a regular, consistent basis. And doing the schoolwork making those phone calls, getting the consents and everything is all limited by not enough public health nurses to do all of the work. We didn’t get any more staff; actually, we lost staff because staff were pulled to run 811, COVID swabbing and COVID vaccinations. And we didn’t have the space to immunize 5 000 kids three times in our baby clinics because people were still having babies and babies still needed all those appointments. We tripled and quadrupled our workload with absolutely no help from our upper management in terms of hiring casuals or providing support.”…*.(Provider level Participant, Initial Interview#6)
	More time to deal with more kids overdue for HPVV and other vaccines, so more needles at a time	Negative	*“We don’t have time necessarily to track down some of those students or their parents for consent. For private. And they have three classrooms now to deal with, not just one. Sheer numbers have increased. That has not really enhanced our ability to get these kids immunized. It’s put up a wider, bigger barrier. The other thing is that we are still working with COVID at this point. And we are still catching up on other programs. Capacity, there is minimal capacity before, and now our capacity has decreased even more.”…*.(Provider level Participant, Initial Interview#10)
	More human resources are required to implement different programs due to varying local capacities.	Negative	*“If we are offering COVID vaccine to that school-age population, that we’re doing that at a totally different time than we’re offering those school-based programs- the HPV, Hep-B, meningococcal. We’re really working hard to separate that programming even though it takes a lot of human resources to be able to implement two different programs primarily at the same time. Once those COVID vaccines started to run, we’ve been consistently running clinics for those vaccines, which is taking up a lot of our time, and a lot of our human resources and in rural, we do not have a COVID-19 immunization team separate than the public health nurses. I don’t think some areas understand that it is the same nurses that are doing school programming, COVID programming, all the Child Health Clinic programming, and all the influenza programming. We don’t have separate teams to do that. We are depending on the same nurses, the same FTE, to do it all. Just that as a system, how we approached administering it was inconsistent based on local ability to either complete the work or not based on the pandemic. And that is because the pandemic impacted local areas of the province differently at any given time.”……*(Provider level Participant, Initial Interview#7)
	Provider Fatigue and Burnout	Negative	*“Resources. Basically, it feels like our workload has doubled. Because we are the main suppliers of the COVID vaccine to the public. That was put on us and our staff to deliver. During the heavy part of it, we were offered and did have some additional immunizers to help us do all that. Currently, those have all been removed and taken away. We are back down to our original staff. And we are still expected to deliver the new program, the COVID program, our school program, our child health conferences. Everything is to go forwards with our original staff. Hence, fatigue and stress, increase in illness, we’re exhausted.”…*(Provider level Participant, Initial Interview#4)
	Lack of Recognition	Negative	*“But I would like to think that for most people, hopefully, the health system and Public Health and the vaccines that we provide to the kids and the COVID vaccines that were developed so quickly and that everyone tried to get into arms so quickly. Hopefully, was a good news story. I know Public Health. Everyone knows who’s Public Health during a crisis. It’s the story of SARS and everything. It needs a good crisis to make people aware of Public Health, but then, it fades away pretty quickly.”…*(System level Participant, Followup Interview#4)
	Staff reallocation and work redistribution	Negative	*Also, many people were focused on the COVID vaccine. So, not just the HPV vaccine but also other immunization coverages were impacted. The demand was there, but the demand for the workforce was in other places. Healthcare workers and public health nurses had to be deployed in other sectors, so that has definitely had an impact …* (System level Participant, Initial Interview#6)
	Testing and contact tracing for other infections halted: Outbreak of other infections, e.g., syphilis.	Negative	*“STIs were not tested, and we did not have time to follow up the cases and the contacts, so we have now too many cases of syphilis and other STIs that were not tested, and they spread. So, that’s another side of looking at it, if something happens in the future, how the public health staff will be able to carry on their local, routine services, Communicable Disease Control, Immunization, the Public Health Inspectors carrying on their public health visits, inspections and everything. So much got lost, not only immunization; our immunization rates have dropped, syphilis is out of control, HIV. We’ve had significant increases in cases of HIV, it’s appalling, actually.”…*(System level Participant, Initial Interview#15)
	Difficult catchup due to a huge backlog	Negative	*“We did actually have to shut our school-based programming down for a year which definitely put us behind. Last year, last spring, we made a big push to try to get caught up on our school-based programming. So, right now, this year, we are doing grades 6, 7, 8, and a little bit of grade 9. Just to try and catch up for that year, year and a half, that we did miss people in getting the series completed. We had kids fall through the crack, I’ll say, with moving and being transient and just missing them during those school years. We had a larger portion of students that were not attending the class that were doing online schooling. And so, we have such a backlog in our public health office visits that we weren’t able to do school-based out of those clinics, so I think it’s just really that backlog that we’re still trying to catch up. We’re a lot closer than we were last year, but we’re still playing catch up right now”……*(System level Participant, Initial Interview#7)
	Vaccine Equipment Handling & Storage	Negative	*“One of the barriers is that in our offices, we are storing a lot more needle supply and alcohol swabs, all those tools that we use and some of those are for mass clinics and some of them are for schools. One of the barriers is that we’re storing both of them in our offices and lots of time there is a lot of supplies that we’re are trying to climb over and figure out which one is going to the school and which one is going to the mass clinic. That is a little bit of a barrier, is being able to store and handle all the equipment we need.”…*.(Provider level Participant, Initial Interview#8)
Vaccine Skepticism	More anti-vaxers: More vaccine hesitancy in parents and more anxious kids	Negative	*“I know the nurses that they’re finding they’re dealing with much more high anxiety kids. They are having to take more time with the children in distraction or stress-reducing activities for immunization. They are also noticing more vaccine hesitancy, parents wanting their children immunized at the offices versus the school. And nurses find there is more hesitancy about vaccines. Since the uproar with the COVID-19 vaccines and some of the conspiracy theories that have been out there, and some parents are applying that to all vaccines.”……*(Provider level Participant, Initial Interview#11)
	More refusal of vaccines	Negative	*“And then, we could see is more like public skepticism with regards to public health since COVID. Increased misinformation on social media leading to vaccine hesitancy. We definitely have seen some parents who I feel would have accepted the vaccines before deciding to decline them now*. *Some parents stopped immunizing completely, once COVID vaccines came out, they lost a lot of trust in the system. “Well, if they’re forcing COVID vaccine on us are they are forcing this? why?” You know, so they had a lot more questions. We did have some people stop immunizing completely to everything, not just HPV.”…*.(Provider level Participant, Initial Interview#2)
	More public health clinic appointments as opposed to school-based immunization	Negative	*“I don’t know if this is just a local nuance, we’re also seeing a lot more parents requesting their children to be immunize at the office. We have actually increased the number of clinic appointments available because we are finding now, too, I think partially due to the mistrust, more families are preferring to bring their child to clinic rather than having them immunized in the school. I don’t know if that’s still because they worry that if the parent isn’t with them that we will secretly stab them with COVID vaccine, but we are seeing more families asking if they can book their child in.”…*.(Provider level Participant, Initial Interview#4)
	Relationship between school staff and PHNs challenged: Hesitant resumption of former school-based needle program.	Negative	*“Do you know what, I would say pre-COVID, probably a lot better. I think the relationship was, well, number one, the longer that nurse is in that school, the better that relationship is with that school. I 100% believe that you need, whether it be with the client or whether it be with another system like the school, you need to develop that relationship with that school. That relationship is very fragile, so it has to develop not only from the director level, the ED levels, and the school board levels, and it has to develop from up there down. We work hard at developing that relationship so we’re supportive of each other. But we’re guests going into that school, but we still need to be able to do your programs. Obviously, COVID put a lot of barriers to that, our school immunization program and even now, schools, I think, are mostly over their reluctance to have people coming into their schools, but that did impose quite a significant barrier during COVID for sure.”…*.(Provider level Participant, Initial Interview#13)
	Trust Issues: Mistrust to both Public Health and Health Systems	Negative	*“Every school is a challenge for a different reason. It’s too hard of a question to ask because the principle of public health, you’re taking it down to the very basic immunization, which is a task. The trust that the public has in us has been — lost, we lost trust with the public with COVID and that has spilled over into regular immunizations. That trust we have; it isn’t there as much as it used to be. It’ll come back, and it’s going to take a couple of years.”…*.(System level Participant, Initial Interview#3)
The COVID-19 Pandemic: An Example	COVID as an example of HPVV Advertisement: Provincial Education or Awareness Campaign	Positive	*“Immunization as a whole, coming out of the pandemic, immunization became a much more controversial topic maybe during the pandemic, or just there’s more awareness around the topic of immunization coming out of the pandemic, and so, HPV as well as immunization as a whole we’ve really probably need some of those public campaigns and some of that messaging that’s very in the public but with correct information. Not forceful information but supportive information. And with the HPV vaccine. Just education and really kind of visually appealing kind of campaigns and a bit more understanding about that particular immunization and just maybe revisiting some of the early campaigns that were done. Through education, we need to let them know what it means, and it doesn’t have to do anything with their sexual activities, just a preventive program down the line. When they become sexually active, they could be exposed to a virus that could ultimately cause cervical cancer.”…*.(System level Participant, Initial Interview#11)
	Technology can help us	Positive	*“I think on the flip side, there’s people that are more in tune with immunizations, right? Because of what we’ve gone through. So, I think our teams have seen a different way of how we can do things differently, how technology can help us. How we can more modernize population in public health services in a different way using different technologies. So, you know, and also using different scopes of practice to enhance our human resource capacities. So, I think we’ve learned a lot through COVID and can definitely take those things forward.”…*.(System level Participant, Initial Interview#9)
	Practise of Infection Control Guidelines: Positive Behaviour Impact	Positive	*“I think infection control, cause that’s something that was a little bit different compared to pre-COVID is more attention to either the mask, how important is handwashing, and disinfecting before and after. I think those things are a lesson learned going in now, compared to before the infection control guidelines. There’s a lot of safety practices that became second nature with the covid clinics, which I think were described as the best practices in schools that maybe weren’t always being executed that we can bring forward.”……*(System level Participant, Initial Interview#6).
	COVID-Vaccine Related Success: Vaccine Hesitant turned Vaccine Inclined	Positive	*“I think on the flip side, there are people that are more in tune with immunizations, right? Because of what we’ve gone through. So, maybe people that were hesitant before are more inclined, so I think that’s a positive. I think our teams have seen a different way of how we can do things differently, and how technology can help us. How we can more modernize the population in public health services in a different way using different technologies. So, you know, and also using different scopes of practice to enhance our human resource capacities. So, I think we’ve learned a lot through COVID and can definitely take those things forward.”……*.(System level Participant, Initial Interview#12)
	Mode of Consent Changed to accommodate COVID-19-related restrictions	Positive	*“During COVID-19, because we weren’t going into the schools, or they were closed or whatever, I actually had a lot come in prior to school starting, or they would come to my office. I, fortunately, was kept up with all my school immunizations. I didn’t fall behind, as I know some schools did. The consent process, if they were brought into the clinic, I discussed it with the parent at that time and they signed, and I just did right in my office. Then when it was lifted, and we could go back into the schools, then I would go, then the information was just sent out as regular letters or the consent form, the information was just sent out with the child and then returned back to the school.”……*.(Provider level Participant, Initial Interview#6)

^Negative: Impeded HPVV uptake. Positive: likely enhanced HPVV uptake^.

#### The COVID-19 pandemic impact on school-based HPV immunization programme: negative impact

*(1) Resources Scarcity*

All the stakeholders expressed concerns and frustration with the lack of resources. Understaffing, time constraints, backlog, workload, staff reallocation and work redistribution were considered factors originating directly due to the lack of human resources. These factors led to inconsistent local response across regions of SK during the COVID-19 pandemic based on the local ability, which led to difficult catchup on immunization, and challenges of vaccine handling and storage. Providers voiced concerns about all efforts to address the COVID-19-related work and vaccine rollout with little to no plan to carry out at least some of the routine public health activities and services. Deprioritizing other public health services resulted in outbreaks of infectious diseases, such as HIV and syphilis, which are already at higher rates across SK, and complete neglect of Health Promotion.

STIs were not tested, and we did not have time to follow up the cases and the contacts, so we have now too many cases of syphilis and other STIs that were not tested, and they spread. So, that’s another side of looking at it, if something happens in the future, how the public health staff will be able to carry on their local, routine services, Communicable Disease Control, Immunization, the Public Health Inspectors carrying on their public health visits, inspections and everything. So much got lost, not only immunization; our immunization rates have dropped, syphilis is out of control, HIV. We’ve had significant increases in cases of HIV; it’s appalling, actually.… . (System-level Participant, Initial Interview#3)

*(2) Vaccine scepticism*

Stakeholders expressed concerns that they observe more vaccine hesitancy among parents and that children are more anxious about immunization at school or when brought to public health clinics or mass immunization centres. This increased vaccine hesitancy has resulted in more interrogation for other routine vaccines, lower return of consent forms, more refusal of vaccines, and more parents wanting to be present with their child (ren) at the time of immunization. Therefore, more of these parents chose to book an appointment with public health clinics to bring in their child instead of seeking the same vaccine through the school-based immunization programme. Public health clinics are over capacity because they are set up mainly for preschool immunization and other programmes. It is difficult to accommodate the population from school after missing the opportunity. Stakeholders also discussed challenges navigating through school staff to deliver school-based HPV immunization programmes as the COVID-19 pandemic-related disruption of the school-based vaccine programmes disrupted the long-standing relationships between school staff and PHNs. As a result, there is the hesitant resumption of former needle programmes in schools, posing barriers to already overworked staff dealing with huge backlogs and playing catchup. Stakeholders believed that public mistrust of public health and the health system vaccine had catalysed vaccine scepticism due to multiple restrictions proposed by public health.

Again, the next big one was the trust/mistrust with the public health system, or the health system in general. Lessons for me though, too, not with regards to students at all is that, us as a program are very flexible. When we talked to people trying to get consents like you get the idea that they feel like the government is trying to control them. We’ve heard a few things that now they’re thinking that HPV vaccine is a new vaccine, so they don’t trust it. It’s something they were familiar with before, like the old diphtheria and tetanus and stuff that families are used to. So, there’s more distrust in the HPV vaccine.… *… …* . (Provider level Participant, Initial Interview#5)

#### The COVID-19 pandemic impact on school-based HPV immunization programme: positive impact

*The COVID-19 Pandemic: An Example*

All Stakeholders considered adopting strategies adopted during the COVID-19 pandemic for mass immunization and infection containment. Stakeholders suggested the same strategies for vaccination against HPV through public messaging and provincial and educational campaigns. The efforts invested in addressing the COVID-19 pandemic through technology have led us to upgrade and modernize public health. The COVID-19 pandemic proved that public health could be flexible in adopting a hybrid way of collecting consent for vaccines from parents by switching from school-based immunization to in-clinic. This adaptation provides the idea that a hybrid approach could result in sorting consent and being able to immunize. Moreover, during the pandemic, the rigorous use and practice of the Infection Control Guidelines became second nature in clinics, resulting in a positive behavioural change. Finally, many stakeholders considered that the COVID-Vaccine-related success turned many vaccine-hesitant into vaccine inclined. Therefore, they deemed that the COVID-19 pandemic can be an example to guide day-to-day public health practice in many dimensions.

#### Current mandate across Saskatchewan to reach HPV vaccine coverage targets

*Alternative Vaccine Clinics*

Stakeholders across SK reported different and related plans to catch up with immunization based on their local ability. The plans for immunization catchup included mass clinics, community clinics, drive-through immunizations, and additional and extended-hours clinics to reach the HPV vaccine and other vaccine coverage targets that were neglected during the COVID-19 pandemic. Additionally, stakeholders also mentioned plans for more trips to school and mass clinics, with some schools catching up with immunization that is more classroom-focused as opposed to grade-focused. Stakeholders from some regions also plan to immunize in “Blitz” and then transition to “Strategic” because they believe what works for one area does not necessarily work for others, as the number needed to be immunized larger than the provider pool.

Hybrid Types of Immunization Clinics, such as drop-in, school-based, centre-based, physician-based, and pharmacy-based immunizations, are also being considered to reach a mass population. Inside-school and outside-school vaccination connect are planned to get the target audience on immunization using different access points. Interestingly, a stakeholder reported “no plans” to play catch up in their area as they could keep up with vaccination because the region had an ability, based on their locality, to decide on their own. They did not suspend school-based immunizations. The stakeholder called this an anomaly as the COVID-19 pandemic did not impact that area from keeping up with immunization. Please refer to Table V for a summary of the current mandate to reach HPVV coverage targets: themes, subthemes and exemplar quotes.

I know that we’re a little bit of an anomaly, as certainly in rural health because we did make the decision that we were not going to suspend these school-based immunizations during the pandemic. We actually didn’t have any of them that were missed, so we don’t feel that we have to catch up. Part of our rational for that was, we said, ‘We don’t want to come out of this pandemic only to have children —’ I mean, HPV isn’t going to be a pandemic or an epidemic, but we didn’t want to come out of the pandemic with an epidemic of measles or an epidemic of mumps or an epidemic of whatever because we didn’t keep up with these. So, we just felt that that was an important priority. As far as the COVID-19 having an impact on us keeping up with those immunizations, that is not the case here.…*……*(Provider level Participant #10)

**Table V.** Current mandate across Saskatchewan to reach HPV vaccine coverage targets: categories, themes and exemplar quote.

Current Mandate Across Saskatchewan to Reach HPV Vaccine Coverage Targets			
Themes	Sub-themes	Explanation	Exemplar Quote
Alternate Vaccine Clinics	Mass Clinics/Community Clinics	No school-based flu or covid clinics.	*“So, we will have to plan more school-based clinics for these children, and we are also, for the other vaccines, which are not school age vaccines, the flu and the covid. We are encouraging parents to send their children to the mass clinics that we are organizing in the communities. That’s another issue. We cannot go into the school to offer flu clinics or covid clinics. As I said, if we leave everything else and put all of our efforts into school-based immunization, we will still need a lot of time to catch up for those schools because, as I said, we don’t have enough staff to immunize all the children, who are the three categories that we had mentioned: the children who have already missed the vaccine, children who are due now, and the children who will become due in the due process in the coming months.”…*.(Provider level Participant, Initial Interview#2)
	Alternate Vaccine Clinics: Additional and Extended-Hour Clinics	Tracking age groups missed & putting together corrective action plans.	*“Yeah, both offering additional clinics at the public health offices but also making sure we kind of know what age groups were missed in the last couple of years or maybe haven’t had that opportunity. Our public health nurses do have a very good tracking mechanism to kind of know what schools they went into and where they have not and just offering additional clinics within the schools. We had talked about once we get through flu season even trying just to book some additional casual resources to catch up both on our syphilis work and also to address some of our vaccine work and corrective action plans.” …* .(System level Participant, Initial Interview#6)
	Drive Through Immunization	School-based Immunization turned into Drive through Immunization	*“We had to change things up very, very quickly. When the pandemic first hit in March of 2020, we already had our plans to go into school in April, but then we couldn’t do that. We actually quickly flipped, and we set up a drive-thru immunization — we were able to get one of our arenas and it worked really well, actually. It is something that I think we should maybe try to do again in the future because — again, the kids have to come to us, they have to have a vehicle. We had some who biked, but again, you generally need to have a parent or somebody to bring you.”……*.(Provider level Participant, Initial Interview#11)
	More Trips to School to CatchUp for Missed Opportunity	Playing Catchup: More Grades to Track, More Trips to School and More Clinic Appointments	*“When we went into schools — so normally, we’re doing grade sixes and grade eights and this year each nurse had to check their grade six and seven, grade eight and grade nine classrooms. We were going into the grade above to try and catch anybody who we missed. We’re doing more trips to schools, maybe, than what we did before to try and do those catch ups. We have actually increased the number of clinic appointments available because we are finding now, too, I think partially due to the mistrust, more families are preferring to bring their child to clinic rather than having them immunized in the school. I don’t know if that’s still because they worry that if the parent isn’t with them that we will secretly stab them with COVID vaccine, but we are seeing more families asking if they can book their child in. So, we’ve increased appointments that way.”…*(Provider level Participant, Initial Interview#4)
	Strategic CatchUp: Classroom-focused vs grade-focused	Playing Catchup: More Nurses, More Trips to School and Mass Clinics	*“A lot of places I think are doing each classroom now instead of just focusing on the suggested Grade 1,6, and 8. So Grade 1 to 12, and going in one to two times per month to try and catch these places up. Other places are utilizing … like in the bigger centres, they’re utilizing two to four public health nurses going in as a group. And playing catch up that way. Trying to do mass clinic with some schools that are very far behind. How successful has this been, I don’t know. To be honest, I’d actually have to ask that question and I’m not measuring the success for that as well at this point.”*(System level Participant, Initial Interview#7)
	Blitz then Strategic	What works for a certain area works for them.	*“Yes. I described the one where we did have the blitz. I think that was summer 2021, and then beyond that, I think it’s, each school trying to, and each team that serves that school is trying to be creative. So, looking to see how they can decoy more days, right? Larger teams on the same day or what works for that particular school because they no longer just going in to immunize sixes and eights now, they’re going in to immunize six to eight, nine, ten, eleven. And so, the number of those need to be immunized is much larger than the provider pool is, or at least smaller if we were talking about the additional other asks around delivery. So, it’s very team. That team to that school, and trying to find a path forward. Yeah, so right now we’re pushing hard to get things to be a little bit more strategic, but I’d say that’s the approach at the moment as we transition.”*.(Provider level Participant, Initial Interview#13)
	Hybrid Types of Immunization Clinics	To Reach a Mass population, different clinic types were set up	*“We can be very efficient with mass communication, immunization strategies. We can use technology to help with consents with booking with pre-booking, appointments to make it very convenient for the end user. We could do hybrid types of immunization clinics. We can do drive through, drop-in, school-based, center-based, physician-based, pharmacy-based immunizations to reach a mass population.”*(System level Participant, Initial Interview#9)
	Inside School, Vaccination Connect And Outside School Vaccination Connect	Getting the target audience on board using different access points	*“I know I’ll use Linus and they do phone calls; they try to catch them and schedule them when they go into their school visits. So, those would be their main two strategies is to try to connect with them outside of school and arrange a follow-up, and, or connect with those that have missed whether in school vaccinating the current grouping.” … …*(Provider level Participant, Initial Interview#8)
Anomaly	COVID-19 did not impact	We kept up with immunization uptake	*“I know that we’re a little bit of an anomaly, as certainly in rural health because we did make the decision that we were not going to suspend these school-based immunizations during the pandemic. We actually didn’t have any of them that were missed, so we don’t feel that we have to catch up. Part of our rational for that was, we said, “We don’t want to come out of this pandemic only to have children —” I mean, HPV isn’t going to be a pandemic or an epidemic, but we didn’t want to come out of the pandemic with an epidemic of measles or an epidemic of mumps or an epidemic of whatever because we didn’t keep up with these. So, we just felt that that was an important priority. As far as the COVID-19 having an impact on us keeping up with those immunizations, that is not the case here.”…*..(System level Participant, Initial Interview#12)

#### Lessons learnt due to the COVID-19 pandemic

*Gaps in Public Health Systems Planning and Operationalization*

Stakeholders deemed that although they could draw from many lessons, they learnt from the COVID-19 pandemic-related change in public health operations. One critical lesson learnt from the pandemic was that everyone at all levels of the public health and health system realized that serious gaps existed in the public health and health system planning and operationalization. During the COVID-19 pandemic, resources in all capacities were stretched thin. We realized how fragile our public health system is that it lacks a contingency plan in its day-to-day operations and does not have a solid plan to follow and policy to enact to respond to emergencies. The stakeholders expressed frustration that even the H1N1 pandemic was a big lesson that could lead us to formulate a robust emergency preparedness plan, but that was not the case.

Stakeholders articulated that many immunization-related and interrelated issues arose during the COVID-19 pandemic due to a lack of a robust plan to deal with emergencies. These issues ranged from not having a working policy for the mature minors’ consent to immunization hype to deprioritizing *everything* but COVID. They discussed how they felt and struggled when they had to work to get the immunization done at the first opportunity. As to them, failure to do so might mean they might never get it again, or at least it would be difficult to do that then. Please refer to Table VI for a summary of lessons learnt due to the COVID-19 pandemic: themes, subthemes and exemplar quotes.

**Table VI.** Lessons learnt during the COVID-19 pandemic: themes, sub-themes, and exemplar quote.

Themes	Sub-themes	Explanation	Exemplar Quote
Gaps in Public Health Systems Planning and Operationalization	Lack of Contingency Plan	We should have a Plan B	*“A lesson learned is that first of all, we should have a plan B, so that our children, not only school-based immunization, but the childhood immunizations don’t suffer if we are facing something like covid in the future. We should have a plan B and we should have enough staff or personnel that if something like covid happens in the future, the child immunization and the school-based immunization do not suffer. I don’t know how that will be accomplished, as we did through covid, most of the covid or pandemic related work is taken up by other public health staff and the public health staff could go carry on their local work, because it’s not only the vaccination. We are probably in a crisis situation, probably with syphilis and other STIs because we didn’t have the time because covid testing pressure other STIs were not tested and we did not have time to follow up the cases and the contacts, so we have now too many cases of syphilis and other STIs that were not tested and they spread. So, that’s another side of looking at it, if something happens in the future, how the public health staff will be able to carry on their local, their routine services?”……*.(System level Participant, Initial Interview#1)
	Fragile Public Health System	Prioritize COVID over everything or anything.	*“Though another lesson learned is that how fragile our current public health system is. And its resources. That it was unfortunate throughout that period that we had to prioritize COVID and COVID vaccine over school-based immunization because it was really ethically difficult. For both the public health staff and the administration staff to have to make those choices and prioritize one thing over another that are equally important.”…*..(System level Participant, Initial Interview#10)
	Get it at The First Opportunity.	If you do not optimally utilize the first opportunity, you might never get it again.	*“Get it at the very first opportunity, I guess. Like dont you know, be prepared going in. Make sure you have all your work done before going in, because you might not get another opportunity going back. Right. Like there are some kids, cause some kids just fall. There are just three or four kids before we got to see them and if we’re not ready then you’re not getting that kid immunized cause they’re there so rarely.”…*..(System level Participant, Initial Interview#8)
	Lack of Working Policy Around Mature Minor	A way around when the consent form is not returned	*“Secondly, the whole mature minor piece that came more to the forefront during COVID would really like that to be further discussed and apply it more liberally in grade 8. If kids do not bring their content forms back. Yeah, that’s what I can think of what we’ve learned from COVID.”…*..(System level Participant, Initial Interview#15)
	Gaps in Public Health Personnel Planning	Public Health Personnel Planning: Only A Wishful Thinking	*“Covid has revealed that we are lacking in public health personnel planning. I usually say we don’t even have the critical mass of the public health staff to provide routine services because of the shortage of so many public health nurses. On top of that, if something like covid comes, the staff is so overworked that nothing can be done. I usually say that we are our worst enemies in public health because we keep on working. We don’t complain; we just keep on working, we get together and complaint to each other and then just resume work … The staff, the public health nurses, who mainly provide school-age immunization were too busy doing covid-related activities, not only vaccination but case and contact follow-up, they were so busy that covid was the main barrier in the last two years. It has overshadowed all the other barriers. It’s also made the issue of staff shortage, and human resource shortage because we did not have enough personnel to do covid and school immunization at the same time. So, lessoned learned: go with the flow. I don’t know. There was nothing we could have done anyway. In a perfect world, I think what I would have liked to see was the key programs and that would have meant extra public health nurses to cover the regular programming while everybody else focus on the pandemic. But that never did happen anyway, so that’s wishful thinking.”…*.(System level Participant, Initial Interview#1)
	Mistrust of Public Health and Health Systems	COVID Vaccine Mandate and some people’s Reaction: *You are not telling me what to do*	*“Again, the next big one was the trust/mistrust with the public health system, or the health system in general. Lessons for me though, too, not with regards to students at all is that, us as a program are very flexible. When we talked to people trying to get consents like you get the idea that they feel like the government is trying to control them. We’ve heard a few things that now they’re thinking that HPV vaccine is a new vaccine, so they don’t trust it. It’s something they were familiar with before, like the old diphtheria and tetanus and stuff that families are used to. So, there’s more distrust in the HPV vaccine. Everything maybe has to do with the policy- the vaccine passport, they have to have been vaccinated to go to the restaurant, to go to the plane. Some other public health mandate came in because those people may be didn’t like how the way how that happened. They wanted to freedom. They want it to be not at all you need a COVID vaccination or not. It’s small population of those who are saying, “You are not telling me what to do so if you are telling me then I am not even going to do what I want, what I was supposed to do.” I think it’s mainly came out of those vaccine passports, vaccine mandates. The mandate made them mad.” …*.(Provider level Participant, Initial Interview#12)
	Targeted Strategies Based on Local Ability and Variation	A quasi-strategic way to work on the catch-up cohorts at the network level based on the variation	*“I think these trying to look for any window of opportunity in a quasi-strategic way to work on their catch up cohorts. And that’s very much driven at the network level, trying to do it as close to each school and each cohort as possible because we have a lot of variability, and the schools, right. Got some very privileged schools where that parents are upset that we can’t get them in right away to the other clinics, the ones where families are really appreciate a school-based program because getting to the clinics isn’t there so they’d really like to see that kind of model, and they’ve got others where there’s a lot of complex conversations around immunizations overall. So, I’d say the response on this one is it’s, we’re trying to develop strategies as close to the particular cohort who’s behind as possible, and that’s a lot when we have so many schools.”………*..(System level Participant, Initial Interview#9)
	Health Promotion Neglected: Long-Term Plan Needed	Health Promotion Already an Uphill Battle	*“People are just over public health. They don’t want any more immunizations; they don’t want to even talk about how we’ve lost a lot of our health promotion thanks to COVID because everybody had to focus on the COVID response and the whole concept of response is exact opposite of what health promotion is. We have not been able to do health promotion for the last couple years. It was already on a lesser level of intensity, lesser level of involvement within the health care system overall, there isn’t a ton of dollars attached to health promotion, so we don’t have the amount of stuff, we don’t even have nursing resources, so it was already an uphill battle and then taking those resources and putting it to the COVID response has resulted in a lack of health promotion because of having to deal with COVID.”*(System level Participant, Initial Interview#13)
	Long-Term Consequences of Temporary Policy	Pandemic Emergency Policy Proved: A Misfit Policy	*“I think one of the lessons learned, again, is COVID-19 around the school-based immunization programming was, it was a policy that came from the ministry that parents had to be present if the student was going to receive COVID-19 vaccine in the school, which created all kinds of barriers for those people who couldn’t take time off work, couldn’t be at the school, there was some who didn’t have transportation. All kinds of reasons. And that’s not the way that we’ve ever done it before, so I think that that has created a negative consequence and it has a created a bit of mistrust, which is very unfortunate.”…*.(System level Participant, Initial Interview#15)
	Renewed Emphasis and Need for Patient-Centered Approach	Consent Sorting is a Nightmare	*“Oh, consent sorting is a nightmare. It’s one of the most time-consuming problematic issues that comes up is about how, as we talked about earlier, how disruptive that is. How the schools loathe it, the families loathe it, it’s ridden with the ability for consent to get lost, it doesn’t allow—it depends on literacy, it depends on time availability, familiarity. It’s certainly problematic and it would be really nice if we could find a way that was more client centered but also a less of a burden to both the public health side as well as the education side.”*(Problem level Participant, Initial Interview#9)

Stakeholders were frustrated and stated that the COVID-19 pandemic had renewed an emphasis on lacking public health personnel planning. The stakeholders were demoralized by the public loss of trust in public health and the health system due to the proposed restrictions and temporary misfit policies. They cited long-term consequences of short-term policies that misfit with the local abilities across SK as local abilities vary widely in terms of the location of the region (e.g north, rural VS Saskatoon and Regina) and the population they serve. Stakeholders suggested tailored strategies to cope with inconsistent local abilities instead of a provincially dictated one size fits all strategy. Stakeholders emphasized the need for long-term sustainable plans to promote and sustain health promotion—an already uphill battle! Stakeholders highlighted a clear need for practicing a long-suggested “Patient-Centered Approach” to provide routine services and a plan on similar lines for emergency services.

Though another lesson learned is that how fragile our current public health system is. And its resources. That it was unfortunate throughout that period that we had to prioritize COVID and COVID vaccine over school-based immunization because it was really ethically difficult. For both the public health staff and the administration staff to have to make those choices and prioritize one thing over another that are equally important.….(System level Participant, Initial Interview#4).

## Discussion

The study overall sought to gain an in-depth understanding of perspectives on the uptake of HPVV at three levels (patients, providers, and system). This paper reported the following aspect of the study: stakeholders’ perspective on HPVV uptake and also presented findings about the COVID-19 pandemic-related disruptions of the school-based HPVV programme regarding the scope of problems posed and the current mandate to reach HPVV coverage targets.

The study’s main findings are (1) stakeholders perceive that (a) information, awareness and education and (b) vaccine-related logistics are significant in the uptake of HPVV. (2) Stakeholders reported scarcity of resources and vaccine scepticism as the most significant problems posed by the COVID-19-related disruption. In contrast, stakeholders suggested that the COVID-19 pandemic can be an example to guide day-to-day public health practice in many dimensions. (3) Stakeholders planned alternate vaccine clinics and routes to catch up with immunization to reach target coverage rates. They reported that the biggest lesson learned from the COVID-19 pandemic was the serious gaps in public health systems planning and operationalization related to routine services and emergency management.

The study findings agree with the literature that determinants of HPVV immunization uptake are broader in context and difficult to encompass comprehensively. The determinants vary on the bio-psycho-social- environmental- and -political axis. Our study corroborated the factors on HPVV uptake we had previously summarized in our review from across English Canada (Khan et al., 2023) in the context of SK. The study findings align with the literature that HPVV uptake is largely hindered by vaccine attitudes driven by either lack of awareness (Cartmell et al., 2018) information and literacy (Alhusayn et al., 2022; Fernandes et al., 2018) or its adequacy (Lubeya et al., 2022). The study adds to the literature by highlighting the context-specific barriers and facilitators to the uptake of HPVV at three different levels across SK in an attempt to provide views from multiple prescriptive.

The study advances our understanding of the link between the role of bio, psycho-social, environmental, and political determinants and HPVV uptake. Literature resonates with our findings concerning HPV-related knowledge, attitude and belief (Rubens-Augustson et al., 2019; Wilson et al., 2021). Our study findings, however, emphasize that vaccine-related logistics could be equally important, if not more. The study findings suggest that emphasizing COVID-19-related services and suspending all other public health and health systems contributed to challenges in HPVV efforts. This study supports the literature articulating the need for robust emergency preparedness plans and policies that can allow for engagement with emergencies while maintaining other routine public health and healthcare functions to continue (Haldane et al., 2021; Margherita et al., 2021)

Most findings from the initial interviews data set were confirmed with the findings from the follow-up interviews, document analysis and literature. Stakeholders agreed there had been significant erosion of trust in public health and the health system (Hurt, 2022) at large due to some short-term misfit policies during the COVID-19 pandemic, and it will take some time to regain the trust (Confusing COVID-19 advice is undermining public trust; here’s how to restore it, 2020; Henderson et al., 2020). Stakeholders resonated on the idea of periodic education (Fernandes et al., 2018; Tung et al., 2016) and thought of reoffering HPVV to high school youth and possibly universities. Literature, however, supports the implementation of universal HPVV programmes (Salvadori, 2018).

Stakeholders strongly agreed that unambiguous language should be used in introducing HPVV (Dempsey & O’Leary, 2018). Vaccine communication (Michel & Goldberg, 2021; Rubens-Augustson et al., 2019) either verbal or written, via immunization packages should be clear. The vaccine information sheet and consent form should be made (a) simple, less text heavy with a low reading scale, (b) available in different languages, (c)highlight cancer prevention focus, (d) address age appropriateness and related concerns and (e) a user-friendly source that could facilitate and guide parents’ decision-making around HPVV.

Some points of disagreement were found among stakeholders when school spacing for vaccine clinic setup (Enskär et al., 2023) was discussed, as this factor varied based on the area the participant represented. Nuances were also observed in the follow-up interviews as stakeholders highlighted the importance of school vaccine education in the context of the health promotion model (García-Toledano et al., 2022). They believed that a deliberate effort to connect with the channels of communication youth use (Arede et al., 2018) is critical to HPVV effective uptake.

Findings highlighted that while there are guidelines for HPVV uptake by all population types (i.e., special populations with certain diseases or conditions and populations whose immunization record is not certain), there is no working policy or plans for HPVV catchup for migrants who fall outside the routine HPVV programme for equitable HPVV coverage (Rubens-Augustson et al., 2019). While our study supports the literature that an age limit to seeking HPVV through the publicly funded programme is certainly a barrier (Giede et al., 2010) to its uptake by all population subgroups, we did not find any cultural-related factors (Rubens-Augustson et al., 2019) to be a barrier to HPVV uptake.

A major limitation of our study is that we could not assess current area-based coverage rates of HPV immunization of grade sixers (age 9). Therefore, we could not study disparities in the uptake of HPVV all across SK or study regions with lower coverage rates for interviews to explore barriers and facilitators in the uptake of HPVV. While specific findings may not be generalizable beyond the study context, the thematic areas of importance to HPVV may resonate elsewhere, and they can guide thinking about creating robust vaccination programmes in other jurisdictions.

## Conclusion

The study findings suggest that successful vaccination strategies must rely on combination approaches that address the needs of different populations and subgroups. HPV-related literacy and vaccine-logistics are equally important and should be reconsidered especially in the context of hybrid digital and analogue world. We recommend a person-centred approach to enhance HPVV uptake using the determinants of HPVV uptake our study highlighted. For example: educating parents on dimensions of individual needs, including giving the right information, handling misconceptions/myths, and addressing specific questions, concerns, or fears. Systems-level thinking approach that centres the patient and family is needed in which we need to look at all levels of the systems to understand and improve uptake rates.

Also, this study warrants sustainable preparation and practice of emergency preparedness plans and policies with continuous audit and feedback loops to avoid neglecting health promotion goals in the times like the COVID-19 crisis. Future research may fruitfully study and update factors of HPVV uptake across regions during emergencies and normal times.

## Supplementary Material

Interview guide 1_Provider level_clean.docx

Authors Bio.docx

Interview Guide 3_Provider+System Level_clean.docx

Interview Guide 2_System level_clean.docx
