# Supplementary material for: Stakeholders’ perspectives on barriers to and facilitators of school-based HPV vaccination in the context of COVID-19 pandemic-related disruption: a qualitative mixed methods study
Source: Int J Qual Stud Health Well-being. 2023 Dec 20;19(1):2295879. doi: 10.1080/17482631.2023.2295879 (PMC10763868; doi:10.1080/17482631.2023.2295879)
Supplement: Interview guide 1_Provider level_clean.docx [file ZQHW_A_2295879_SM3341.docx]

**Provider-level Interview Guide**

**Initial Interviews**

Assessing what providers of HPV vaccines identify as barriers and facilitators in providing HPV immunization in Saskatchewan -- revealing Provider-Level factors.

**Part 1(A): Situational/setting the stage questions.**

| In this section, we will be asking descriptive elements about the operationalization of the HPV immunization program by providers and managers. |
| --- |

1. What is your position in the HPV immunization program in your health authority? What area in Saskatchewan do you work/serve?

2. How long have you been in your position?

3. What is your health authority's overall mandate of HPV immunization?

**Part 1(B):** Providers’ Perspectives on HPV Immunization Programs

| In this section, we will be asking some questions to reveal providers’ perspectives on HPV immunization programs in their respective public health units. |
| --- |

1. What is your perspective in general on HPV immunization programs?

2. How does your health authority devise strategies and decisions about HPV immunization

programming? Specifically, for example,

(a) What divisions and personnel are involved in planning HPV immunization services?

(b) How often are service plans revisited?

(c) What types of information are used in HPV immunization service planning?

**Part 2(A): Probing/Open-ended questions**

| In this section, we will be exploring provider-level barriers in the operationalization (planning, programming, roll-out and delivery) of HPV school-based immunization in the province of Saskatchewan. |
| --- |

In general,

(a) how do parents/guardians respond to being offered the HPV vaccine for their child?

(b) In your opinion, what could be some of the concerns people have about deciding against getting the HPV vaccine?

(c) Do these questions, concerns and reasons differ between male and female children?

(d) How would you respond to those questions, concerns, and reasons?

(e) What could be done to enhance your ability (as providers) to respond to questions, concerns, and reasons for declining the vaccine?

**Part 2(B):** Providers’ Perspectives on School-based HPV Immunization Programming and Delivery in the Province of Saskatchewan **Prior** to the COVID-19 Pandemic

| In this section, we will further explore the school-based HPV immunization programming and programming delivery in the province of Saskatchewan **prior** to the COVID-19 pandemic. |
| --- |

(a) What kinds of things do you think the school-based HPV immunization program does particularly well when offering the HPV vaccine?

(b) What program logistics (i.e., capacity, vaccine supply, timing) might be a barrier to youth being immunized with the HPV vaccine?

(c) What are your thoughts on the Vaccine Information Sheet provided to parents/guardians on the HPV vaccine?

(d) What do you think of the distribution and return process for the **consent form** and accompanying program materials?

I. Any opinion on how the consent process be improved?

(e) What opportunities are there to improve the school-based HPV immunization program overall?

I. What is already being planned and implemented?

II. What are some barriers to making these improvements?

**Part 2(C): Scope** of the problem posed **due to** COVID-19-related school-based immunization program disruptions.

| In this section, we will be asking about the school-based HPV immunization programs that are going on in Saskatchewan today as of the 2021-2022 school year to examine the scope of the problem posed due to covid-19 related disruptions. |
| --- |

1. How has COVID-19 impacted the school-based HPV immunization program?
2. How has the school-based HPV immunization program changed due to COVID-19?

a. I asked you about the process prior to COVID-19, specifically the consent process, how the immunization is administered, and the barriers to immunizations.

i. How has the consent process changed due to COVID-19?

ii. How has HPV vaccine administration changed due to COVID-19?

iii. How have the barriers to immunizations changed due to COVID-19?

**Part 3(A): Systematic prompting questions**

| In this section, we will be exploring provider-level barriers and facilitators in the operationalization of HPV school-based immunization in the province of Saskatchewan. |
| --- |

1. What is working well in HPV immunization planning and implementation?
2. What is not working well, and who/what is being missed?
3. In your opinion, are there specific sub-populations that you think have lower HPV immunization coverage?
4. What strategies do you propose to enhance the uptake of HPV immunization in the future?
5. In your opinion, who should implement the strategies you proposed to enhance the uptake of HPV immunization and how?

*The additional information section is meant to allow participants to add something relevant to the topic that we have missed or they would want to touch base on*

**Additional Information**

| In this final section, we will be asking if there is anything else to be shared about school-based HPV immunization in the province of Saskatchewan. |
| --- |

As it is evident that I am interested in HPV vaccination uptake factors (i.e. barriers and facilitators), in particular, and best vaccination programming and practices.

1. Is there anything else pertinent to this topic that I did not discuss and you would want to add - you are welcome to do so.
2. There are a variety of ways to help improve immunization coverage rates - any general opinion on this matter?

|  |
| --- |
