# Supplementary material for: Stakeholders’ perspectives on barriers to and facilitators of school-based HPV vaccination in the context of COVID-19 pandemic-related disruption: a qualitative mixed methods study
Source: Int J Qual Stud Health Well-being. 2023 Dec 20;19(1):2295879. doi: 10.1080/17482631.2023.2295879 (PMC10763868; doi:10.1080/17482631.2023.2295879)
Supplement: Authors Bio.docx [file ZQHW_A_2295879_SM3340.docx]

**Authors Bio**

Amal Khan^1^

Biography: Amal Khan is a physician and population health specialist. She is an elected member of the board of directors at Basic Income Canada Network. She completed her medical degree from Combined Military Hospital, Lahore and her Master's in Community and Population Health Sciences at the University of Saskatchewan, Canada and is now a Ph.D. Candidate at the same institute. She studied population health dynamics as a functional proxy of equity in society, economy, politics and beyond. She is working on a large national (Canada-wide) project under the Urban Public Health Network with a lens of equity, contributing to monitoring Population Health and Local Public Health Action in Urban Canada. Her research journey has shaped her belief and driven her passion, where one cannot keep oneself from the action. Her research interests include Health Equity, Social Determinants of Health, Immigrant and Refugee Health, Preventable Cancers, Mixed methods designs, Qualitative and Quantitative Data Collection & Analysis, and Medicine and Society. Amal is a member of the Canadian Cancer Society Research Institute. She is currently working to co-develop a rapid diagnosis program for the early detection of upper gastrointestinal cancers with an equity lens at the Saskatchewan Cancer Agency.

Sylvia Abonyi^2^

Biography: I am the Canadian-born daughter of an immigrant and refugee, a wife, and the mother of two. As an educator, health researcher, and citizen in Canada, I teach, learn, work, and live on traditional Indigenous territories that include treaty lands, unceded lands, and Métis homelands. I pay my respects to the First Nation, Métis, and Inuit ancestors. Trained as an anthropologist, I conduct community engaged research with First Nation and Métis communities and peoples, primarily from northern Saskatchewan. Recent studies focus on healthy aging in place, tuberculosis, sleep health, as well as heart and lung health. Together with Indigenous community partners, I am also part of national and provincial teams revealing experiences of covid-19. In addition to my position in the department, I am research faculty with the Saskatchewan Population Health and Evaluation Research Unit (www.SPHERU.ca), an interdisciplinary group of researchers from the Universities of Saskatchewan and Regina whose collective focus is on advancing health equity through the creation of new knowledge, independent policy analysis, and collaborative research with policymakers and communities.

Cory Neudorf^3^

Biography: As a public health physician and epidemiologist, he has spent his career devoted to health equity and public health advocacy. For more than 20 years, Neudorf served as chief medical officer for the former Saskatoon Health Region. Recently, he served as the interim senior medical health officer with the Saskatchewan Health Authority. Neudorf is also involved with public health at the national level as the current president of the Urban Public Health Network of Canada, former president of both the Canadian Public Health Association and the Public Health Physicians of Canada, liaison member with the Regions for Health Network (WHO Europe) and advisor for the Public Health Agency of Canada. To him, Public Health provides the chance to influence health and other policy and get more upstream on issues rather than individual patient issues, which appeals to him. He describes himself as a health geek. It’s an apt description of a man who has spent the past 24 years dedicated to promoting and protecting the health of people in communities in Saskatchewan and around the world. He has travelled to northern Saskatchewan, as well as Eastern Europe and Central Asia – nearly 20 countries all together – in the interest of global health.

Sandro Galea^4^

Biography: Sandro Galea, a physician, epidemiologist, and author, is dean and Robert A. Knox Professor at Boston University School of Public Health. In 2015, he became the youngest public health dean in the country, assuming leadership of the Boston University School of Public Health. One of the most widely-cited scholars in the social sciences, Galea has published more than 950 scientific journal articles, 70 chapters, and 19 books. He has published extensively in the peer-reviewed literature about the social causes of health, mental health, and trauma. His research has been principally funded by the National Institutes of Health, Centers for Disease Control and Prevention, and philanthropic foundations. Galea has received several lifetime achievement awards for his research, including the Rema Lapouse Award from the American Public Health Association and the Robert S. Laufer, PhD, Memorial Award from the International Society for Traumatic Stress Studies. Galea holds a medical degree from the University of Toronto, graduate degrees from Harvard University and Columbia University, and an honorary doctorate from the University of Glasgow. Prior to his appointment at Boston University, Galea served as the Gelman Professor and Chair of the Department of Epidemiology at the Columbia University Mailman School of Public Health.

Shahid Ahmed^5^

Shahid graduated from Dow Medical University of Health Sciences, Karachi, in 1996. He completed his training in Internal Medicine and Medical Oncology at the Long Island Jewish Medical Center, Albert Einstein College of Medicine, New York, in 2003. He completed his Doctor of Philosophy in Community and Population Health Science from the University of Saskatchewan in 2016. He is a fellow of the American College of Physicians and the Royal College of Physicians and Surgeons of Canada. Shahid joined the Saskatoon Cancer Center in 2003 as a medical oncologist. His area of interest is breast and gastrointestinal oncology. In addition to patient care, he is committed to medical education and research. He is an active member of various local and national committees that are involved in patient care, medical education, and research. He has served as a continuing professional development educator for the Royal College of Physicians and Surgeons of Canada. He holds several research grants and actively engages in collaborative research and clinical trials. Shahid has presented his works at several international meetings, published more than 70 articles in peer-reviewed journals, and author of several book chapters.
