# Supplementary material for: Stakeholders’ perspectives on barriers to and facilitators of school-based HPV vaccination in the context of COVID-19 pandemic-related disruption: a qualitative mixed methods study
Source: Int J Qual Stud Health Well-being. 2023 Dec 20;19(1):2295879. doi: 10.1080/17482631.2023.2295879 (PMC10763868; doi:10.1080/17482631.2023.2295879)
Supplement: Interview Guide 3_Provider+System Level_clean.docx [file ZQHW_A_2295879_SM3339.docx]

**Provider and System level Interview Guide**

**Follow-up Interviews**

##

**Part 1:**  Awareness, Information and Education -- revealing System & Provider Level Factors

| In this section, we will be discussing ways to create awareness of, information on and education about HPV infections and HPV vaccines for enhancing HPV vaccine uptake rates. |
| --- |

Q1. Provider & system-level participants emphasized the Patient-Centred Approach in HPV vaccine delivery and administration in phase-1 interviews. Based on our phase-1 interview data analysis, the central theme is educating decision-makers in consenting or declining HPV vaccine (e.g. parents) and vaccine receivers (e.g. youth).

(a) Using the Patient-Centred Approach, how can we give the right information to parents and youth while handling misinformation and addressing parents' concerns about HPV immunization?

(b) How can we develop an avenue for one-on-one interaction (either face-to-face or virtual) between Public Health Nurses and Parents?

(c) What would be some of the strategies to create one-on-one interaction opportunities between Public Health Nurses and migrant parents?

*Migrants (immigrants and refugees)

Q2. During COVID-19 Pandemic, many kids (and mature minors) reached out to public health staff to self-consent for getting COVID-19 vaccines for themselves -- this has renewed an emphasis on youth education on infectious diseases and immunization. Also, not all parents and kids (youth) know that there is an age limit for seeking the HPV vaccine free of cost in Saskatchewan until the 27th birthday. If you realize the HPV vaccine is still available, you can get it free.

(a) What avenues do you think of in educating or informing youth on how to get access to the HPV vaccine if their parents have declined on their behalf in the past?

(b) Do you suggest changing the consent policy for mature minors around HPV immunization? Under what conditions or circumstances? How would you propose to respond to any negative reaction to such a move?

(c) Do you think periodically reoffering the HPV vaccine again in high school or university would be a way to enhance HPV uptake rates?

(d) Do you have any other ideas?

**Part: 2** Vaccine Logistics

| In this section, we will explore ways to **amend** the mode of distribution of immunization material through school-based programs, content on the vaccine info sheet, and potential use of immunization data-based and school databases. |
| --- |

All parents expressed the need for more than one option to receive vaccine information material (vaccine info sheet and consent form). This need is because relying solely on a child to bring in the material and return the consent form is challenging. Almost all the system and provider-level participants also agreed that collecting back consent forms is the **biggest challenge** and one of the reasons for non-optimal HPV vaccine uptake rates.

Q1. What is your opinion on using a combination method (hybrid approach) in distributing vaccine information packages to parents through school-based immunization programs?

*Combination method can include any of the following and beyond Paper copy + Email reminder with Electronic consent form *OR* Paper copy + Text reminder with a link to the consent form OR Paper copy + A reminder on the School app: Edsby.

Q2. In addition to parents, many provider and system-level participants agreed that the vaccine info sheet and consent form are difficult to understand because of technical language and the perception that the wording is too “politically correct.”

(a) How can we make the vaccine information sheet a user-friendly guide for parents that they find easy to read and understand?

Q3. Since the HPV vaccine is labelled as a sex-related vaccine, provider-level participants voiced concerns that on the vaccine information material, a disproportionate emphasis has been placed on HPV being a sexually transmitted infection versus HPV-related cancer prevention. This disproportionate emphasis poses further challenges to the already prevailing misconception. Therefore, shifting the focus from STI discussion to better-promoting cancer protection is required. According to the participants, it puts off parents when they read the information material, especially in the context that a younger child (grade sixer) is bringing it home.

(a) Do you agree? Whether Yes/No, please explain.

(b) How can this improvement be made/achieved best?

Q4. What are your thoughts on linking Panorama and the School data system for sending out consent forms and vaccine info material to (a) all students eligible for the HPV vaccine and (b) catch up with those who are behind in immunization due to any reason?

*Reasons: Truant - chronic absenteeism, sick on the day of vaccination, new admission to the school, frequent movers, home schoolers, online learners, etc.).
