# Supplementary material for: Stakeholders’ perspectives on barriers to and facilitators of school-based HPV vaccination in the context of COVID-19 pandemic-related disruption: a qualitative mixed methods study
Source: Int J Qual Stud Health Well-being. 2023 Dec 20;19(1):2295879. doi: 10.1080/17482631.2023.2295879 (PMC10763868; doi:10.1080/17482631.2023.2295879)
Supplement: Interview Guide 2_System level_clean.docx [file ZQHW_A_2295879_SM3338.docx]

**System-level Interview Guide**

**Initial Interviews**

**HPV Immunization Survey Under the National CPAC UPHN Barriers to HPV Immunization Project**

Assessing the operationalization of HPV vaccine programs delivery in Saskatchewan and the impacts of COVID-19 on HPV school-based immunization and investigating the current mandate in place to reach HPV vaccine coverage targets -- revealing System-Level Factors

**Part 1(A): Situational/setting the stage questions.**

| In this section, we will be asking some questions to reveal the perspectives of the system-level stakeholders about HPV immunization programs in their respective public health units. |
| --- |

1. Which region of Saskatchewan do you work in? What is your work title/ position?

2. What is your perspective in general on HPV immunization programs?

3. How does your health authority devise strategies and decisions about HPV immunization

programming? Specifically, for example:

(a) What divisions and personnel are involved in planning HPV immunization services?

(b) How often are service plans revisited?

(c) What types of information are most often utilized in HPV immunization service planning?

**Part 2(A): Operationalization of School-based HPV immunization programs prior to COVID-19**

| In this section, we will be asking about descriptive elements of the school-based HPV immunization programming in the province of Saskatchewan prior to the COVID-19 pandemic. |
| --- |

1. (a) Describe the process of HPV immunization delivery in schools prior to the COVID-19 pandemic.

(b) How was consent collected from parents/guardians?

(c) Who administered immunizations in schools?

(d) What months were HPV immunizations generally given, or did they change year to year?

1. Do you believe the HPV immunization program before the COVID-19 pandemic was effective? If yes/not, why, and how?
2. Describe any barriers to HPV immunization in schools prior to the COVID-19 pandemic.
   1. Examples: Consent process? Religious/moral objections? Vaccine hesitancy in general?
3. We are interested in how public health works with schools to coordinate and administer HPV immunizations. How did this work prior to COVID-19?

a. How did they communicate and work with each other?

b. Who were the representatives from each sector?

| In this section, we will be further exploring the school-based HPV immunization programming and delivery in the province of Saskatchewan prior to the COVID-19 pandemic. |
| --- |

1. (a) What kinds of things do you think the school-based HPV immunization program does particularly well when offering the HPV vaccine?

(b) What do you think of the distribution and return process for the consent form and accompanying program materials? What are your thoughts on the Vaccine Info sheet?

(c) In your opinion:

I. What opportunities are there to improve the school-based HPV immunization program?

II. What is already being planned and implemented?

III. What are some of the barriers to making improvements?

**Part 2(B): Scope of the problem posed due to COVID-19-related school-based immunization program disruptions.**

| In this section, we will be asking about the school-based HPV immunization programs that are going on in Saskatchewan today as of the 2021-2022 school year to examine the scope of the problem posed due to COVID-19-related disruptions. |
| --- |

1. How has COVID-19 impacted the HPV immunization program?
2. In your opinion, how has the school-based HPV immunization program **changed** due to COVID-19?

*a.* *I asked you about the process prior to COVID-19, specifically the consent process, how the immunization is administered, and the barriers to immunizations. Now in relation to COVID-19, in your opinion:*

*i.* *How has the consent process changed?*

*ii.* *How has HPV immunization administration changed?*

*iii.* *How have the barriers to immunizations changed due to COVID-19?*

*In this section, we will be asking about the* ***current mandate*** *in place in Saskatchewan to reach HPV vaccine coverage rates.*

**Part 3: Current mandate in place to improve HPV vaccine coverage rates via school-based immunization programming.**

1. (a) What are the current strategies in place or being used to catch up with youth who may have missed HPV immunizations since March 2020?

(b) How successful have these been?

(c) How are you measuring success?

1. (a) What are the plans for future school-based HPV immunization programming?

(b) How will these be accomplished?

3. (a) What are some lessons learned from COVID-19 regarding school-based immunization

Programming?

(b) Have there been any *positive/negative* unintended consequences? If so, what are they?

*The additional information section is meant to allow participants to add something relevant to the topic that we have missed or they would want to touch base on*

##

##

##

**Additional Information**

| In this final section, we will be asking if there is anything else to be shared about school-based HPV immunization in the province of Saskatchewan. |
| --- |

1. Is there anything else you want to add about the impact of COVID-19 on school-based HPV immunizations in Saskatchewan? You are welcome to do so!
2. Is there anyone else we should be talking to about this to learn more about the Saskatchewan experience in relation to school-based HPV immunization? If so, would you be able to share their contact information with me?
